# Co-regulation analysis of closely linked genes identifies a highly recurrent gain on chromosome 17q25.3 in prostate cancer

**DOI:** 10.1186/1471-2407-8-315

**Published:** 2008-10-30

**Authors:** Raquel Bermudo, David Abia, Berta Ferrer, Iracema Nayach, Alberto Benguria, Ángel Zaballos, Javier del Rey, Rosa Miró, Elías Campo, Carlos Martínez-A, Ángel R Ortiz, Pedro L Fernández, Timothy M Thomson

**Affiliations:** 1 Department of Molecular and Cell Biology, Instituto de Biología Molecular de Barcelona, Consejo Superior de Investigaciones Científicas, Barcelona, Spain; 2 Institut d'Investigacions Biomèdiques August Pi i Sunyer, Barcelona, Spain; 3 Bioinformatics Unit, Centro de Biología Molecular Severo Ochoa, Consejo Superior de Investigaciones Científicas and Universidad Autónoma de Madrid, Cantoblanco, Madrid, Spain; 4 Department of Pathology, Hospital Clínic de Barcelona, Barcelona, Spain; 5 Department of Immunology and Oncology, Centro Nacional de Biotecnología, Consejo Superior de Investigaciones Científicas and Universidad Autónoma de Madrid, Cantoblanco, Madrid, Spain; 6 Institut de Biotecnologia i Biomedicina and Department of Cell Biology, Physiology and Immunology. Universitat Autònoma de Barcelona, Barcelona, Spain; 7 Genomics Unit, Centro Nacional de Investigaciones Cardiovasculares (CNIC), Madrid, Spain

## Abstract

**Background:**

Transcriptional profiling of prostate cancer (PC) has unveiled new markers of neoplasia and allowed insights into mechanisms underlying this disease. Genomewide analyses have also identified new chromosomal abnormalities associated with PC. The combination of both classes of data for the same sample cohort might provide better criteria for identifying relevant factors involved in neoplasia. Here we describe transcriptional signatures identifying distinct normal and tumoral prostate tissue compartments, and the inference and demonstration of a new, highly recurrent copy number gain on chromosome 17q25.3.

**Methods:**

We have applied transcriptional profiling to tumoral and non-tumoral prostate samples with relatively homogeneous epithelial representations as well as pure stromal tissue from peripheral prostate and cultured cell lines, followed by quantitative RT-PCR validations and immunohistochemical analysis. In addition, we have performed *in silico *colocalization analysis of co-regulated genes and validation by fluorescent in situ hybridization (FISH).

**Results:**

The transcriptomic analysis has allowed us to identify signatures corresponding to non-tumoral luminal and tumoral epithelium, basal epithelial cells, and prostate stromal tissue. In addition, *in silico *analysis of co-regulated expression of physically linked genes has allowed us to predict the occurrence of a copy number gain at chromosomal region 17q25.3. This computational inference was validated by fluorescent *in situ *hybridization, which showed gains in this region in over 65% of primary and metastatic tumoral samples.

**Conclusion:**

Our approach permits to directly link gene copy number variations with transcript co-regulation in association with neoplastic states. Therefore, transcriptomic studies of carefully selected samples can unveil new diagnostic markers and transcriptional signatures highly specific of PC, and lead to the discovery of novel genomic abnormalities that may provide additional insights into the causes and mechanisms of prostate cancer.

## Background

In a search for new molecular markers capable of conferring more specificity and sensitivity to prostate cancer (PC) diagnosis, many laboratories in the past few years have applied transcriptomic profiling analyses, which have unveiled new differentially expressed genes in non-tumoral and tumoral prostate tissues. Expression microarrays have also been used to identify profiles characteristic of metastasic disease [1], prostate intraepithelial neoplasia (PIN) [2], and subgroups of tumors with distinct outcomes [3], including a 5-gene model predictive of recurrence [4]. Several of these studies compared tumor tissue with benign hyperplastic tissue, or with non-tumoral prostate tissues that were not precisely characterized in terms of location or epithelial representation. Therefore, the outcomes of these analyses, although identifying genes whose expression patterns were most strongly altered in PC, were possibly biased because the comparisons included tissues of diverse histological or embryological origins, or with undefined epithelial and stromal contents. The high degree of tissue heterogeneity of the prostate represents a challenge for molecular studies of PC, and must be taken into account when performing high-throughput analyses. The representation of each cell type within a given sample determines the overall expression profile, which makes it difficult to compare prostate samples which have very different epithelial and stromal contents. One study addressed this issue by applying *in silico *corrections to compensate for variable epithelial representations in different samples [5], whereas other studies resorted to laser microdissection and *in vitro *linear amplification [6].

Great efforts have also been dedicated to elucidate the molecular bases of prostate carcinoma, which are beginning to provide important mechanistic insights into some of the key events in PC initiation and progression. For example, the inactivation of the PTEN and p53 tumor suppressor genes have been shown to play major roles in the initiation of PC [7]. Also, fusions of TMPRSS2 (transmembrane protease, serine 2) with different members of the ETS transcription factors family are likely relevant initiating factors in this neoplasia [8]. Moreover, genomewide analyses of single nucleotide polymorphisms have allowed the identification of polymorphisms and copy number variations associated either with predisposition to prostate cancer in familial clusters [9,10], or with the occurrence and aggressiveness of prostate cancer [11].

In this study, we have performed a transcriptomic study of carefully selected samples with the aim of finding expression profiles characteristic of the different cell type compartments of the prostate, to better understand the molecular events responsible for this malignancy. Our analysis has allowed the identification of transcriptional profiles and new markers characteristic of different prostate compartments, as well as a new highly recurrent gain on chromosome 17q25.3 associated with prostate cancer.

## Methods

### Tissue samples and cell lines

Tissues were procured from untreated patients undergoing radical prostatectomy for clinically localized prostate adenocarcinoma at the Hospital Clínic of Barcelona. Samples were obtained after informed consent by the patients and approval by the Institutional Ethics Committee. Tissue fragments from tumoral and non-tumoral areas were embedded in OCT, snap-frozen, and stored at -80°C at the tumor bank of this institution (Table 1). Non-tumoral samples were completely devoid of cells or glands with neoplastic appearance upon histologic examination of serial sections corresponding to the processed tissues, and are hereafter called normal samples. The rest of the specimen was routinely formalin-fixed and paraffin-embedded. HeLa and RWPE1 cells (ATCC, Manassas, VA) were grown in DMEM (PAA, Ontario, Canada) supplemented with 10% FBS or keratinocyte serum-free medium (KSFM; Gibco, Carlsbad, CA), respectively. Primary cultures (PC1 and PC2) were derived from prostatectomies in which the adenocarcinoma was macroscopically detected. Fibroblast-depleted explants were grown for 4–5 weeks in KSFM supplemented with 10^-11 ^M 5-α-dihydrotestosterone.

**Table 1:** Clinico-pathological characteristics of prostate samples

			WHOLE TISSUE							MICRODISSECTED (μD)	
	
CASE^a^	GLEASON SCORE	STAGE	TUMORAL SAMPLES	%EP^b^	%T^c^	NORMAL SAMPLES	%EP^b^	%T^c^	STROMAL SAMPLES	NORMAL SAMPLES	TUMORAL SAMPLES
	
1	6 (3+3)	T2	T1^d, e^	70	40	N1^d, e^	40	0	-	N1-μD^e^	T1-μD^e^
	
2	9 (4+5)	T3a	T2^d^	90	100	N2^d^	50	0	-	-	-
	
3	5 (2+3)	T3a	T3^d, e^	80	85	N3^d, e^	40	0	-	N3-μD^e^	T3-μD^e^
	
4	7 (3+4)	T2	T4^d^	60	80	N4^d^	40	0	-	N4-μD^e^	T4-μD^e^
	
5	7 (4+3)	T2	T5^d^	65	90	N5^d^	45	0	-	-	-
	
6	9 (5+4)	T2	T6^d, e^	80	100	N6^d, e^	40	0	-	N6-μD^e^	T6-μD^e^
	
7	7 (3+4)	T3a	T7^d, e^	80	100	N7^d, e^	55	0	-	N7-μD^e^	T7-μD^e^
	
8	7 (3+4)	T2c	T8^d^	80	100	-	-	-	S1	-	-
	
9	8 (3+5)	T2	T9^d^	80	90	-	-	-	-	-	-
	
10	7 (3+4)	T3a	T10^d^	80	100	-	-	-	-	-	-
	
11	7 (3+4)	T3a	T11^d^	70	80	-	-	-	-	-	-
	
12	7 (3+4)	T3a	T12^d^	50	90	-	-	-	-	-	-
	
13	9 (4+5)	T3a	T13^d^	80	100	-	-	-	-	-	-
	
14	7 (3+4)	T3a	T14^d^	65	90	-	-	-	-	N14-μD^e^	T14-μD^e^
	
15	7 (3+4)	T3a	T15^d^	70	100	-	-	-	-	-	-
	
16	5 (2+3)	T2	T16^d^	80	90	-	-	-	-	N16-μD^e^	T16-μD^e^
	
17	7 (3+4)	T3a	T17^d^	80	95	-	-	-	-	-	-
	
18	8 (3+5)	T2	T18^d^	65	95	-	-	-	-	-	-
	
19	6 (3+3)	T2	T19^d^	70	90	-	-	-	-	-	-
	
20	7 (4+3)	T3a	T20^d^	60	80	-	-	-	-	-	-
	
21	-	-	-	-	-	-	-	-	S2	-	-
	
22	-	-	-	-	-	-	-	-	S3	-	-
	
	-	-	-	-	-	POOL N^d^	45	0	-	-	-

^a^Samples in the same case line were obtained from the same patient.

^b^Percentage of epithelial cells.

^c^Percentage of epithelial cells with morphological features of carcinoma.

^d^Samples used in microarray analysis.

^e^Samples used in Q-PCR analysis

### Total RNA isolation

Microscopical examination of hematoxylin-eosin (H&E) stained sections from frozen tissues was used to assess the percentage of each histological compartment (stroma, total epithelium, normal and neoplastic epithelium) prior to RNA extraction. Normal samples contained an average of 45% epithelium, stromal samples, obtained from non-tumoral areas of the specimens, contained less than 1% epithelium and tumoral samples contained an average of 70% epithelium, being 90% of it neoplastic glands. At least twenty 20-μm cryosections were used for RNA isolation, and the first and the last sections were H&E-stained to monitor for tumoral and normal gland status. RNA from tissues and cell lines was isolated using RNeasy kits with a DNase I digestion step (Qiagen, Valencia, CA), and analyzed on a 2100 Bioanalyzer instrument (Agilent Technologies, Palo Alto, CA) to assess quality and quantity.

### Microarray hybridization and data analysis

The samples used for microarray analyses were grouped in four biological groups: adenocarcinomas (n = 20), normal samples (7 normal samples from 7 prostate cancer patients and a pool of normal prostate tissues, n = 8), and normal-associated stromal samples (n = 3), all of them from the peripheral zone of the prostate, and the cell lines described above (n = 4). Biotinylated cRNA (10 μg) from the 35 samples enumerated above was processed and hybridized to Affymetrix Human Genome Focus Arrays (Affymetrix, Santa Clara, CA). Arrays were scanned at 3 μm resolution in an Agilent HP G2500A GeneArray scanner (Agilent Technologies). Data were RMA-normalized [12], followed by quantile-quantile probe-level between-array normalization [13]. Normalized expression data were analyzed by FADA [14], which applies Q-mode Factor Analysis. Genes were considered differentially expressed between the tumoral and normal groups when their associated *q*-value was below 2.5 × 10^-4^. *Q*-values were computed from t-test *P*-values using the Benjamini-Hochberg step-down false-discovery rate (FDR) algorithm [15]. Normalized expression data were standardized and submitted to UPGMA hierarchical clustering [16]. Microarray data sets from this study are deposited in the Array Express repository under accession number E-MEXP-1331.

### Laser Microdissection

Tissue samples from seven of the patients whose samples had been used in microarray analysis were selected for laser microdissection (Table 1). Eight-μm cryosections were mounted onto plastic membrane slides (PALM, Bernried, Germany), fixed in cold 70% ethanol, stained with hematoxylin, dehydrated, air-dried and stored at -80°C until use. Laser microdissection was performed using the PALM MicroBeam System, with the Laser Microdissection and Pressure Catapulting technology. Approximately 1.2 mm^2 ^of both normal and tumoral epithelium were collected separately from each sample and RNA isolation was performed as above.

### Real-Time RT-PCR (Q-PCR)

Microdissected tissues and 4 paired normal-tumoral samples included in the microarray analysis were used for Q-PCR analyses. Custom-designed TaqMan Low Density Arrays (TLDA; Applied Biosystems, Foster City, CA) contained primers and probes for 45 genes, and the RPS18 gene as the calibrator. One ng of total RNA was used as template for reverse transcription for each replicate of non-microdissected (triplicates) and microdissected (quadruplicates) samples. Q-PCR was performed on an ABI PRISM 7900 HT instrument (Applied Biosystems), and relative quantitation determined by the ΔΔCt method.

### Immunohistochemistry

Tissue microarrays (TMA), were built using a Manual Tissue Arrayer 1 (Beecher Instruments, Sun Prairie, WI), and contained a total of 52 paraffin-embedded tumors, 21 PIN and 40 normal samples, each in duplicated or triplicated cores. Two μm thickness TMA sections were mounted on xylaned glass slides (DAKO, Glostrup, Denmark) and used for immunohistochemistry. Mouse monoclonal antibodies specific for myosin VI (MYO6, clone MUD19; 1/100 dilution) and ephrin type-A receptor 2 precursor (EPHA2, clone D7; 1/50 dilution) were purchased from Sigma (Madrid, Spain) and a rat monoclonal antibody specific for multidrug resistance-associated protein 4 (ABCC4, clone M4I-10; 1/50 dilution) was purchased from Abcam (Cambridge, MA). Immunohistochemistry was performed with the Envision system (DAKO) and developed with diaminobenzidine, after antigen retrieval in a pressure cooker with citrate buffer pH 6 (MYO6) and EDTA pH 9 (ABCC4) or no retrieval (EPHA2). The staining was scored as a percentage of positive cells and its intensity as null (0), weak (1), moderate (2) or strong (3). Differences over 20% in the percentage of positive cells and/or one or more than one degree of intensity were considered as a significant change in expression. Images were obtained in an Olympus BHT microscope (Olympus, Germany) with an Olympus Camedia C-3030 camera. Immunohistochemistry information is MISFISHIE compliant [17].

### *In silico *analysis of chromosomal localizations of coexpressed genes

To determine putative non-random colocalizations of coexpressed genes, we determined the precise genomic localizations of all the genes present on the array (ENSEMBL-NCBI 36 assembly of the consensus human genome sequence). We then determined the groups of four or more consecutive FADA-selected genes that were all either over- or underexpressed in tumoral samples and simultaneously colocalized within a distance of 4 Mb, or less. We modelled the random distribution of over- or underexpressed genes as a Poisson distribution, taking as lambda parameter the product of the number of genes present in the array within the tested region multiplied by the total number of over- or underexpressed genes and divided by the number of genes in the array.

### Fluorescent *in situ *hybridization (FISH)

Tumoral samples from the same 20 adenocarcinoma cases used in the microarray analysis and 8 lymph node metastases were used. From each paraffin-embedded sample, a 2-μm section was obtained for FISH analysis and a consecutive tissue section was H&E-stained in order to identify the tumoral and normal regions to be analyzed. Sections were deparaffinized, washed in 2 × SSC, dehydrated, denatured and hybridized with a BAC probe corresponding to the segment of interest on 17q25.3 (RP11-165M24) and a chromosome 17 centromeric probe (CEP17, Vysis, Des Plaines, IL). Slides were then washed in 0.4 × SSC and in 2 × SSC, counterstained with DAPI II (Vysis) and imaged using an Olympus BX60 fluorescence microscope (Olympus) with the MetaSystems software (MetaSystems, Germany). Signals corresponding to both the RP11-165M24 and the chromosome 17 centromeric probes were scored in 200 non-overlapping nuclei of the tumoral zone and gains were defined in samples where ≥ 10% of the analyzed nuclei presented a ratio between the RP11-165M24 and the reference probe ≥ 1.5. In all cases, a non-tumoral area was analyzed to assess its normal chromosomic status.

## Results

### Transcriptional profiles of tumoral and normal prostate compartments

**Figure 1:**
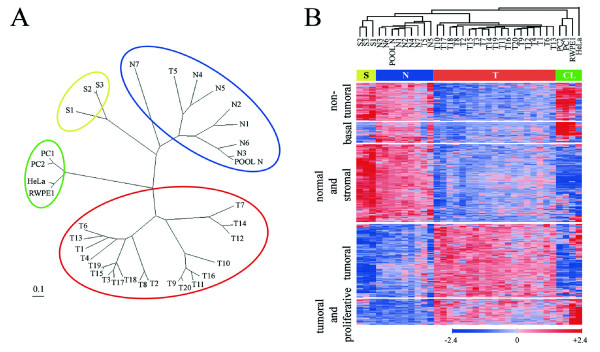
**Graphical representation of microarray data analysis**. A) Unrooted dendrogram showing the clustering of samples into four different classes: normal samples (*blue*), tumoral samples (*red*), stromal samples (*yellow*) and cultured cell lines (*green*). B) Unsupervised hierarchical cluster representation using microarray data for the 318 FADA-selected genes. T, tumoral samples; N, normal samples; S, pure stromal samples; CL, cell lines.

The specific features of our transcriptional profiling included a stringent selection of normal and tumoral prostate tissue samples, based on epithelial representations, the use of pure stromal samples, and the inclusion of four epithelial cell cultures. These cell lines were HeLa cells as a non-prostate epithelial cell line, a normal prostate epithelial cell line (RWPE1) and two prostate primary cultures, the last three with features of basal epithelial cells, as discussed below. The microarray data analysis by FADA permits to identify those genes that most significantly contribute to a given phenotype, and to group the samples according to their degree of biological relatedness [14]. FADA identified 318 genes as those most significantly differentially expressed between normal and tumoral tissues. These genes clustered samples into four groups according to their origin (tumoral, normal and stromal samples, and a group comprising all cultured cells, Figures 1A and 1B), and clustered genes into five functional groups (non-tumoral, basal, normal luminal and stromal, tumoral and tumoral-proliferative, Figure 1B). Of these genes, 134 were overexpressed and 184 underexpressed in tumoral *vs*. normal prostate tissues (Figure 1B, see Additional files 1 and 2). The genes overexpressed by tumors could be further subdivided into two groups according to their coexpression in the different sample groups: a tumoral gene set (99 genes), most expressed in tumoral samples, and a tumoral and proliferative gene set (35 genes) most expressed in both tumoral samples and cultured cells. Similarly, the genes underexpressed in tumors could be subdivided into three subgroups of coexpressed genes: a non-tumoral set (51 genes), expressed at high levels in normal prostate, pure stromal and cultured cell samples, and at significantly lower levels in tumoral samples; a normal luminal epithelium and stromal set (105 genes), highly expressed in normal and stromal samples, but expressed at low levels in tumoral samples or in cultured cells; and a basal epithelial set (28 genes), strongly expressed in normal prostate samples and in cultured prostate epithelial cells, but weakly expressed in tumoral or stromal samples or in non-prostate HeLa cells. The consideration of the latter gene set as characteristic of prostatic basal epithelium relies on the facts that many of its member genes are known markers of basal cells of prostate glands and other stratified epithelia, such as those for tumor protein p63 (TP73L), keratin 5 (KRT5), keratin 7 (KRT7) and keratin 14 (KRT14), or laminin beta 3 (LAMB3) [18,19], and that they lack expression of markers of prostate malignancy such as alpha-methylacyl-coenzyme A racemase (AMACR, [20]) and hepsin (HPN, [21,22]).

### Laser microdissection and Q-PCR validations

**Figure 2:**
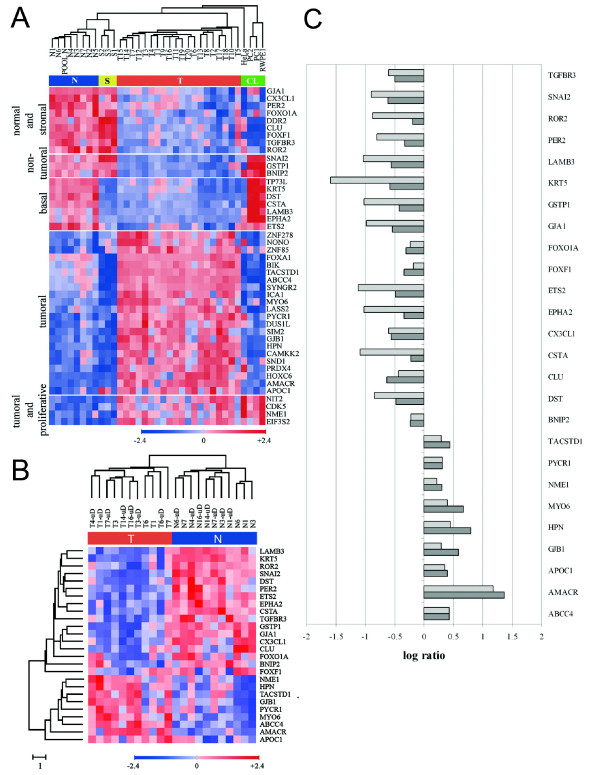
**Quantitative RT-PCR validation of selected genes**. A) Hierarchical clusters built using microarray data corresponding to the 45 genes selected for Q-PCR validation. T, N, S and CL are as described in Fig. 1. B) Hierarchical sample and gene cluster generated with Q-PCR data for genes showing concordance with microarray data (either upregulated or downregulated in both determinations) in 4 normal-tumoral non-microdissected matched samples and 7 normal-tumoral microdissected matched samples. C) Graphical representation of the Q-PCR tumoral/normal ratios for the 26 genes showing the best concordance with microarray data (9 overexpressed and 17 underexpressed in tumoral *vs*. normal samples). Shown are the averages for each of the selected genes of the Q-PCR tumoral/normal ratios for microdissected epithelia (light gray bars) and for whole, non-microdissected tissues (dark gray bars).

As a validation of the microarray expression data, 45 out of the 318 tumoral-normal discriminant genes selected by FADA were further validated by Q-PCR on tumoral-normal paired samples, including whole tissue samples as a technical validation and microdissected tissues in order to assess whether the over- or underexpression of these genes was associated with the epithelial component of the samples. These genes were selected for Q-PCR validation according to their ranking by FADA for discrimination between tumor *vs*. normal prostate samples, and their capacity to correctly cluster samples into the above four categories obtained by microarray analysis (Figure 2A).

For the four non-microdissected paired samples, 1 of the 45 genes analyzed could not be evaluated due to failure of the TaqMan probe to yield reproducible quantitation. Of the 44 remaining genes, 31 (31/44, 70%) presented tumoral *vs*. normal Q-PCR ratios that were similar to, or in the same direction as (upregulated or downregulated) those determined by microarray analysis for the same samples (Figure 2B and 2C). For the 7 microdissected pairs, 43 of the 45 genes could be evaluated, 28 of which (28/43, 65%) yielded tumor *vs*. normal Q-PCR ratios that roughly corresponded to those determined by microarrays for the same samples, 26 of these 28 genes being coincident with the 31 genes validated in non-microdissected samples (Figure 2C). These results reflect the usefulness of selecting samples with special attention to their histological characteristics, but also the necessity of validating microarray data results by complementary approaches such as Q-PCR.

### Immunohistochemical validation of selected new candidate markers of PC

**Figure 3:**
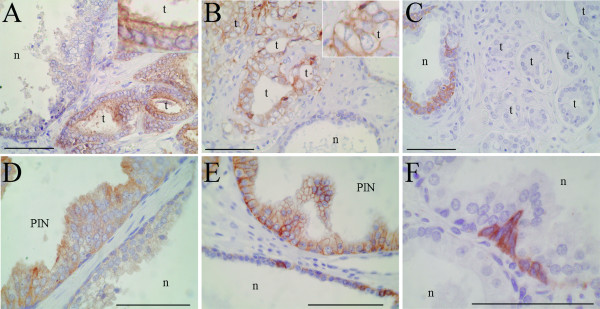
**Immunohistochemical analysis of selected markers**. A, D): Overexpression of myosin VI (MYO6) in tumoral (*t*) and PIN glands, as compared to normal (*n*) glands (inset, higher magnification showing a luminal reinforcement). B, E): Overexpression of multidrug resistance-associated protein 4 (ABCC4) in tumoral and PIN glands (inset in B, higher magnification showing a membrane staining pattern). C) Ephrin type-A receptor 2 precursor (EPHA2) was not detected in tumoral glands, while it was expressed in a subpopulation of basal cells in normal glands (*n*). F) Higher magnification showing a cytoplasmic and membrane staining pattern of EPHA2. Bars correspond to 100 μm.

The expression of proteins corresponding to several of the above defined functional gene clusters was additionally validated by immunohistochemistry on tissue microarrays. A monoclonal antibody to myosin VI (MYO6) stained luminal cells with a homogeneous cytoplasmic pattern, with tumor cells showing frequent apical reinforcement (Figure 3A and 3D). This protein was overexpressed in a majority of prostate tumors (41 of 49 samples in which both tumoral and normal glands were represented in the same cores, 83.6%), and also in a majority of PIN lesions (17 of 21 samples, 80.6%). The multidrug resistance-associated protein 4 (ABCC4) showed a predominant membrane staining pattern (Figure 3B and 3E), and was overexpressed in 82.9% (39 of 47 samples) of tumor cases and in 81.25% (13 of 16 samples) of PIN lesions. Finally, the ephrin type-A receptor 2 precursor (EPHA2), whose transcript was underexpressed in tumoral samples, was undetectable in luminal cells from normal and tumoral glands or stroma, while it was strongly expressed in a subpopulation of basal cells in normal glands (Figure 3C and 3F), consistent with the inclusion of this gene in the basal cell compartment signature.

### *In silico *prediction and experimental validation of a recurrent genomic gain in 17q25.3

**Figure 4:**
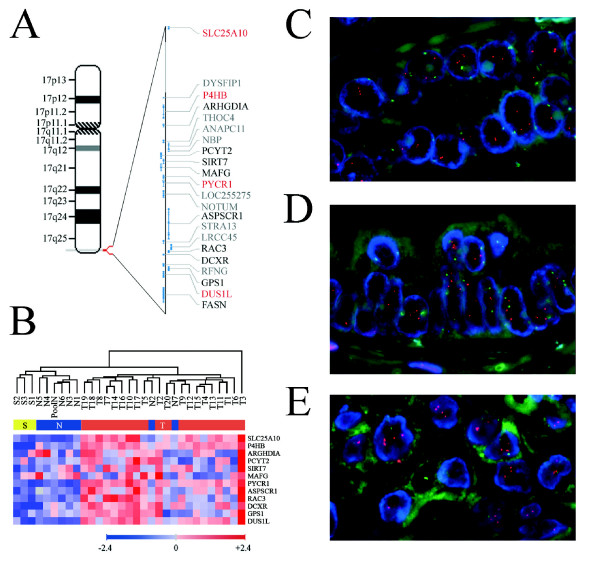
**Identification of a highly recurrent chromosomal gain on 17q25.3 in prostate tumors by transcript co-regulation analysis**. A) Ideogram of chromosome 17 detailing the genes located on 17q25.3 with a corregulated overexpression in prostate cancer, as determined by our microarray analysis (*red*: genes selected by FADA as overexpressed in tumor samples; *black*: genes not selected by FADA but present in the Human Genome Focus microarrays; *grey*: genes not present in Human Genome Focus microarrays). B) Heat map showing the relative expression levels of the genes on 17q25.3 shown in (A). The order of the genes is from centromeric (top) to telomeric (bottom). Fluorescent *in situ *hybridization of prostate tissue samples (C, D and E) hybridized with the centromeric CEP17 probe (*green*) and the 17q25.3-specific BAC clone RP11-165M24 (*red*). Selected representative regions of prostate carcinoma (C) and PIN (D) areas from the same sample, and a lymph node metastasis (E), illustrating copy number gains in 17q25.3. The figures shown are also representative of samples with a 17q25.3 gain.

The analysis of colocalization of genes in adjacent positions in the genome can provide insights into higher levels of regulation that determine the selective coexpression of genes in cancer or putative regions of chromosomic instability [23]. The criteria used in this analysis included a significant association of coexpression for 4 or more FADA-selected genes colocalized within 4 Mb or less in the genome. Our analysis found one cluster of contiguous overexpressed genes with a significant bias in distribution (p = 6.85 × 10^-4^), located on chromosome 17q25.3 (Figure 4A), which included five FADA-selected genes: synaptogyrin 2 (SYNGR2), solute carrier family 25 member 10 (SLC25A10), procollagen-proline, 2-oxoglutarate 4-dioxygenase (proline 4-hydroxylase), beta polypeptide (P4HB), pyrroline-5-carboxylate reductase 1 (PYCR1) and dihydrouridine synthase 1-like (DUS1L). The last four genes colocalize within a segment of less than 0.4 Mb. We re-analyzed the expression profiles of the genes in the 17q25.3 region present in the microarrays used in this study, and found that many of the genes in this region were in fact overexpressed in a significant proportion of tumors compared to both normal and stromal tissues, even if they had not been selected in our original FADA analysis because of their lower discriminant power when taken individually (Figure 4B).

The relatively coordinate overexpression of so many genes, with a tight genomic colocalization to 17q25.3, could be due to genomic amplifications or gains of this region or, alternatively, to the co-regulation by factors acting in *trans*, or by a locus control region effect. In order to address this issue, we performed fluorescent *in situ *hybridization (FISH) on paraffin-embedded samples, corresponding to the same 20 cases analyzed for their transcriptional profiles. The results showed a gain in 17q25.3 (Figure 4C) in a majority of tumors (13 out of 20, 65%), which was never observed in matched normal glands. The median gain observed was 3 copies of the region, which correlated well with the overall levels of overexpression of the genes in this chromosomal region, with ratios generally not above 2-fold (tumoral *vs*. normal tissue). Genetic and phenotypic heterogeneity within tumors is a recurring theme in prostate cancer [24], a possible reflection either of multiclonality or of clonal drift, and it was also observed in this analysis. This heterogeneity was found amongst samples from different cases carrying the alteration, where the percentage of cells with a gain in 17q25.3 varied from 14% to 80%, with a mean of 47.4% nuclei containing the abnormality. Furthermore, the 17q25.3 gain showed a heterogeneous distribution in any given sample, with tumoral zones of the same sample with and without this alteration. Since we could only analyze some regions of the samples, this heterogeneity suggests that the observed prevalence of this segmental copy number gain is most likely an underestimation.

Prostate intraepithelial neoplasia (PIN) lesions could be analyzed near the tumoral zone in four of the tumors that presented this gain. Although the number of PIN nuclei were usually not sufficient for statistic significance, all cases showed a gain in 17q25.3 (Figure 4D), suggesting that this alteration is an early event in prostate cancer. Finally, the frequent finding of this gain in more than 60% (5 of 8) of metastatic samples (Figure 4E), with a mean of 69.4% of nuclei containing the alteration (ranging from 47.5% to 98.5%), indicates that this alteration is conserved during all steps of tumor progression.

## Discussion

Our microarray analysis of highly selected prostate samples, which included normal, tumoral and stromal samples with defined epithelial and stromal representations, and basal epithelial cell lines, has allowed us to identify sets of genes specific of the major cellular compartments in normal and tumoral prostate tissue. Each gene set includes known markers of these compartments, which lends support to our classification, and therefore permits us to infer that other genes in these sets will be relevant in each of these cell types. Amongst the genes included as overexpressed in our prostate tumoral set, there are several well-established PC markers, such as AMACR [20] and HPN [21,22], or previously associated with PC, like those for ectonucleoside triphosphate diphosphohydrolase 5 (ENTPD5), tumor-associated calcium signal transducer 1 (TACSTD1), single-minded homolog 2 (SIM2) or myosin VI (MYO6) [25,26]. Likewise, the tumoral and proliferative gene set includes the gene for cyclin-dependent kinase 5 (CDK5), which has been shown to regulate cell proliferation in thyroid carcinoma [27]. Similarly, several of the genes in the non-tumoral set have been described as selectively suppressed in PC, like those for caveolin-1 (CAV1) [28], caveolin-2 (CAV2), annexin A2 (ANXA2) [29], or glutathione-S-transferase π1 (GSTP1) [30]; some genes such as desmin (DES), fibroblast growth factor 2 (FGF2) or transforming growth factor beta receptor III (TGFBR3) have been described to be predominantly expressed by the stromal compartment [5]; finally, several genes in the basal set are also known markers of basal cells of prostate glands and other stratified epithelia, such as those for tumor protein p63 (TP73L), keratin 5 (KRT5), keratin 7 (KRT7), keratin 14 (KRT14), or laminin beta 3 (LAMB3) [18,19]; the underexpression of these genes in tumoral samples could reflect the loss of this cell layer in prostate tumors. Similarly, our immunohistochemical analyses show that the ephrin type-A receptor 2 precursor (EPHA2), which is classified in this basal gene set, is expressed in a restricted compartment of the basal cell layer. It is also worth noting that all these basal markers are expressed at high levels by our primary cultures, suggesting that they have basal cell features.

Further computational analysis of our FADA-selected genes, in which we applied profiling-based *in silico *predictions of genomic imbalances, have led us to the prediction of a recurrent copy number gain in 17q25.3, a prediction that was subsequently demonstrated experimentally by FISH. This was the most significant genomic imbalance predicted by our analysis, and, given the strong functional significance of the gene sets selected by FADA, we suggest that the 17q25.3 segmental gain may play a relevant role in prostate carcinogenesis. Although comparative genomic hybridization studies have revealed gains in distal segments of 17q in some tumors, including prostate cancer [31,32], they have not been associated with the precise region in 17q25.3 described in our study. Our analysis suggests that this recurrent gain may involve a region as small as 0.4 Mb in size, which may fall below the resolution of genomewide BAC array analysis [33].

Considering only the most significantly biased four-gene cluster on 17q25.3, there are 21 genes in the region comprised between SLC25A10, the most centromeric gene of the cluster, and DUS1L, the most telomeric one, of which 13 are represented in the microarrays used in this study (Figure 4A). Of these genes, of immediate interest in cancer are ARHGDIA, that codes for an inhibitor of GDP dissociation from the ras-like cytoskeleton regulator Rho [34]; ANAPC11, coding for an essential subunit of the anaphase-promoting complex [35]; SIRT7, coding for an homologue of the NAD^+^-dependent histone deacetylase SIRT1 that regulates RNA polymerase II [3]; MAFG, whose product is a basic region leuzine-zipper transcription factor that heterodimerizes with NRF-2 to regulate the transcriptional response to oxidative stress, and also with Fos and JunB [36,37]; and ASPSCR1 (also known as ASPL), a gene that is frequently translocated in alveolar soft part sarcomas and papillary renal cell carcinomas to the chromosome X gene TFE3, causing the increased expression of this transcriptional regulator [38]. Also located in this region, immediately telomeric to DUS1L, is the fatty acid synthase gene (FASN), a well known marker of malignancy and progression in PC [39].

The discovery of gene copy number variations acquires added significance if it is simultaneously correlated with transcriptional profiling, a combined approach that few studies have followed [23,40,41]. Thus, we believe that the relevance of our finding of a novel recurrent gain in 17q25.3 in prostate cancer resides mainly on the facts that it is correlated with coordinate overexpression of genes that are tightly associated with that region, and that it is the most significant copy number abnormality predicted by our approach and that it is a recurrent gain, observed in 65% of primary prostate cancer cases. This frequency is significantly higher than most of the recurrent gene copy number variations reported in association with prostate cancer [11,41-44]. Although gains in 17q25.3 have been reported by other laboratories as part of high-throughput screenings aimed at identifying genomic alterations in association with prostate cancer [41,43], they were not analyzed in detail. Therefore, our identification is the first to directly correlate this tumor-associated abnormality with co-regulation of transcripts encoded by specific genes in this region, applying a reverse-genetics approach that combines transcriptomic analyses, *in silico *predictions and experimental verification by FISH. Finally, the fact that this genetic aberration is detected in PIN and also in metastasis samples is suggestive of its involvement in all the stages of malignant transformation and progression in PC.

## Conclusion

Careful selection of samples with known epithelial and non-epithelial composition, and the inclusion of pure prostate stromal tissues, combined with the application of an inclusive, non-supervised analysis method based on Factor Analysis (FADA), has permitted the extraction of biologically significant sets of genes characteristic of prostate cancer and of the major prostate tissue compartments. Further analysis of transcript levels from these FADA-selected genes, treated as coexpression of closely linked genes, has allowed the identification of a highly recurrent chromosomal gain on chromosome 17q25.3, encompassing 21 genes. This chromosomal aberration is present in 65% of primary prostate cancers and metastases, and also in PIN lesions, making it one of the most frequent genetic abnormalities associated with all stages of malignancy in prostate cancer.

## Competing interests

RB, DA, BF, AB, AZ, EC, CM-A, ARO, PLF and TMT are co-inventors in a patent application related to part of the results described in this manuscript.

## Authors' contributions

RB, PLF and TMT have participated in the design, execution, analysis and writing of all the sections in this report. BF, IN and EC have participated in sample procurement and processing, and in immunohistochemical analyses. DA and ARO have participated in biocomputational analyses. AB, AZ and CM-A have participated in microarray data generation. JdR and RM have contributed to FISH analysis.

## Pre-publication history

The pre-publication history for this paper can be accessed here:



## Supplementary Material

Additional file 1 **Table showing FADA-selected genes information**. Genes selected by FADA analysis as the most discriminant between tumoral and normal prostate samples. Click here for file

Additional file 2 ** Graphical representation of the genes selected by FADA**. Unsupervised hierarchical clusters built with the expression data from the 318 genes selected by FADA as the most discriminant genes between tumoral and normal samples. Click here for file
