# Supplementary material for: Co-regulation analysis of closely linked genes identifies a highly recurrent gain on chromosome 17q25.3 in prostate cancer
Source: BMC Cancer. 2008 Oct 30;8:315. doi: 10.1186/1471-2407-8-315 (PMC2585097; doi:10.1186/1471-2407-8-315)
Supplement: Additional file 1 — Table showing FADA-selected genes information. Genes selected by FADA analysis as the most discriminant between tumoral and normal prostate samples. [file 1471-2407-8-315-S1.pdf]

Supplementary Table 2. Genes selected by FADA as the most discriminant between tumoral and normal prostate samples

GeneID	T-test	q-val	Gene Symbol	Gene Title	GO Biological Process Description	GO Molecular Function Description	GO Cellular Component Description	Pathway
213572_s_at	-6.753	2.213E-05	SERPINB1	serpin peptidase inhibitor, clade B (ovalbumin), member 1	---	serine-type endopeptidase inhibitor activity /// endopeptidase inhibitor activity /// serine-type endopeptidase inhibitor activity	cytoplasm	---
204158_s_at	-5.729	8.585E-05	TCIRG1	T-cell, immune regulator 1, ATPase, H+ transporting, lysosomal V0 subunit A3	ion transport /// cellular defense response /// positive regulation of cell proliferation /// proton transport /// transport /// proton transport	transporter activity /// hydrogen ion transporter activity	plasma membrane /// integral to plasma membrane /// membrane /// integral to membrane	---
213194_at	-6.168	4.441E-05	ROBO1	roundabout, axon guidance receptor, homolog 1 (Drosophila)	chemotaxis /// cell adhesion /// homophilic cell adhesion /// nervous system development /// cell differentiation /// positive regulation of axonogenesis /// development /// nervous system development	receptor activity /// axon guidance receptor activity /// identical protein binding /// protein binding	integral to plasma membrane /// cell surface /// membrane /// integral to membrane	---
203411_s_at	-5.297	1.636E-04	LMNA	lamin A/C	---	structural molecule activity /// protein binding /// microtubule motor activity /// motor activity	nucleus /// intermediate filament /// cytoplasmic dynein complex /// microtubule /// dynein complex	---
201329_s_at	-5.807	7.442E-05	ETS2	v-ets erythroblastosis virus E26 oncogene homolog 2 (avian)	skeletal development /// regulation of transcription, DNA-dependent	transcription factor activity /// sequence-specific DNA binding /// DNA binding /// transcription factor activity /// DNA binding	nucleus	Smooth_muscle_contraction

202464_s_at	-5.034	2.458E-04	PFKFB3	6-phosphofructo-2-kinase/fructose-2,6-biphosphatase 3	fructose 2,6-bisphosphate metabolism /// fructose 2,6-bisphosphate metabolism /// metabolism	nucleotide binding /// catalytic activity /// 6-phosphofructo-2-kinase activity /// 6-phosphofructo-2-kinase activity /// fructose-2,6-bisphosphate 2-phosphatase activity /// fructose-2,6-bisphosphate 2-phosphatase activity /// ATP binding /// kinase activity /// transferase activity /// hydrolase activity /// identical protein binding	---	Fructose and mannose metabolism /// Fructose and mannose metabolism
215127_s_at	-5.066	2.351E-04	RBMS1 /// C2orf12 /// LOC648293	RNA binding motif, single stranded interacting protein 1 /// chromosome 2 open reading frame 12 /// region containing chromosome 2 open reading frame 12; RNA binding motif, single stranded interacting protein 1	DNA replication /// DNA replication /// RNA processing /// regulation of translation /// cytokine and chemokine mediated signaling pathway /// regulation of protein complex disassembly /// protein amino acid phosphorylation /// DNA replication /// protein amino acid phosphorylation /// signal transduction	nucleotide binding /// DNA binding /// double-stranded DNA binding /// double-stranded DNA binding /// single-stranded DNA binding /// single-stranded DNA binding /// RNA binding /// RNA binding /// protein binding /// identical protein binding /// magnesium ion binding /// protein serine/threonine kinase activity /// ATP binding /// nucleic acid binding /// magnesium ion binding /// protein kinase activity /// protein serine/threonine kinase activity /// protein binding /// ATP binding /// kinase activity /// transferase activity	nucleus /// nucleus	---
209272_at	-6.317	3.885E-05	NAB1	NGFI-A binding protein 1 (EGR1 binding protein 1)	transcription /// regulation of transcription, DNA-dependent /// negative regulation of transcription /// regulation of transcription, DNA-dependent	transcriptional repressor activity /// transcriptional repressor activity	nucleus /// nucleus	---
204030_s_at	-6.382	3.490E-05	SCHIP1	schwannomin interacting protein 1	---	---	cytoplasm	---

200984_s_at	-5.554	1.108E-04	CD59	CD59 molecule, complement regulatory protein	immune response /// cell surface receptor linked signal transduction /// blood coagulation /// defense response	protein binding /// GPI anchor binding	membrane fraction /// plasma membrane /// membrane	---
209308_s_at	-6.634	2.510E-05	BNIP2	BCL2/adenovirus E1B 19kDa interacting protein 2	apoptosis /// anti-apoptosis /// apoptosis	GTPase activator activity /// calcium ion binding /// protein binding	nuclear envelope /// cytoplasm /// intracellular membrane-bound organelle	---
201590_x_at	-5.916	6.118E-05	ANXA2	annexin A2	skeletal development	phospholipase inhibitor activity /// phospholipase inhibitor activity /// calcium ion binding /// calcium-dependent phospholipid binding /// cytoskeletal protein binding	soluble fraction /// plasma membrane	Prostaglandin_synthesis_regulation
203323_at	-6.351	3.721E-05	CAV2	caveolin 2	---	protein binding /// protein homodimerization activity	plasma membrane /// integral to plasma membrane /// caveolar membrane /// caveolar membrane /// lipid raft /// perinuclear region /// membrane /// integral to membrane	Integrin-mediated_cell_adhesion_KEGG
203065_s_at	-5.982	5.829E-05	CAV1	caveolin 1, caveolae protein, 22kDa	cholesterol transport /// cholesterol homeostasis	structural molecule activity /// protein binding /// cholesterol binding	Golgi membrane /// endoplasmic reticulum /// integral to plasma membrane /// membrane /// integral to membrane /// caveolar membrane /// caveolar membrane /// lipid raft	Integrin-mediated_cell_adhesion_KEGG /// Integrin-mediated_cell_adhesion_KEGG
201798_s_at	-5.295	1.636E-04	FER1L3	fer-1-like 3, myoferlin (C. elegans)	muscle contraction /// circulation	---	nuclear envelope /// plasma membrane /// integral to membrane /// membrane	---
201136_at	-5.399	1.401E-04	PLP2	proteolipid protein 2 (colonic epithelium-enriched)	ion transport /// chemotaxis /// cytokine and chemokine mediated signaling pathway	protein binding /// ion transporter activity /// chemokine binding	membrane fraction /// endoplasmic reticulum /// endoplasmic reticulum membrane /// plasma membrane /// integral to membrane /// membrane	---

217728_at	-6.577	2.637E-05	S100A6	S100 calcium binding protein A6 (calcyclin)	regulation of progression through cell cycle /// cell cycle /// signal transduction /// cell-cell signaling /// axonogenesis /// cell proliferation /// positive regulation of fibroblast proliferation /// cell cycle	calcium ion binding /// growth factor activity /// protein homodimerization activity /// S100 beta binding /// S100 alpha binding /// calcium-dependent protein binding /// protein binding /// calcium ion binding /// calcium ion binding	ruffle /// nucleus /// nuclear envelope /// cytoplasm /// cytoplasm	Prostaglandin_synthesis_regulation
203729_at	-5.347	1.505E-04	EMP3	epithelial membrane protein 3	development /// cell death /// negative regulation of cell proliferation /// cell growth	---	membrane fraction /// membrane /// integral to membrane	---
221676_s_at	-6.067	5.213E-05	CORO1C	coronin, actin binding protein, 1C	phagocytosis /// signal transduction	actin binding	actin cytoskeleton	---
AFFX-HSAC07/X00351_5_at	-5.296	1.636E-04	ACTB	actin, beta	---	nucleotide binding /// structural constituent of cytoskeleton /// protein binding /// ATP binding /// structural molecule activity /// protein binding	cytoskeleton /// actin filament /// TIP60 histone acetyltransferase complex	Smooth_muscle_contraction /// Smooth_muscle_contraction /// Striated_muscle_contraction
208029_s_at	-6.207	4.242E-05	LAPTM4B	lysosomal associated protein transmembrane 4 beta /// lysosomal associated protein transmembrane 4 beta	---	---	integral to membrane	---
202760_s_at	-6.23	4.242E-05	AKAP2 /// PALM2-AKAP2	A kinase (PRKA) anchor protein 2 /// PALM2-AKAP2 protein	regulation of cell shape	kinase activity /// enzyme binding	membrane	G_Protein_Signaling /// G_Protein_Signaling /// G_Protein_Signaling /// G_Protein_Signaling
203939_at	-5.15	2.046E-04	NT5E	5'-nucleotidase, ecto (CD73)	DNA metabolism /// nucleotide catabolism	nucleotide binding /// 5'-nucleotidase activity /// zinc ion binding /// hydrolase activity, acting on ester bonds /// metal ion binding /// GPI anchor binding /// 5'-nucleotidase activity /// hydrolase activity	membrane fraction /// membrane	Purine metabolism /// Pyrimidine metabolism /// Nicotinate and nicotinamide metabolism
203562_at	-6.61	2.523E-05	FEZ1	fasciculation and elongation protein zeta 1 (zygin I)	cell adhesion /// nervous system development /// axon guidance	protein binding	---	---

218854_at	-5.984	5.829E-05	SART2	squamous cell carcinoma antigen recognized by T cells 2	---	---	endoplasmic reticulum /// membrane /// integral to membrane	---
212190_at	-5.191	1.906E-04	SERPINE2	serpin peptidase inhibitor, clade E (nexin, plasminogen activator inhibitor type 1), member 2	nervous system development /// cell differentiation /// regulation of proteolysis /// regulation of cell migration /// development	serine-type endopeptidase inhibitor activity /// heparin binding /// endopeptidase inhibitor activity /// serine-type endopeptidase inhibitor activity /// heparin binding	extracellular region	---
200824_at	-5.955	5.829E-05	GSTP1	glutathione S-transferase pi	anti-apoptosis /// central nervous system development /// metabolism	glutathione transferase activity /// transferase activity	cytoplasm	Circadian_Exercise /// Glutathione metabolism /// Metabolism of xenobiotics by cytochrome P450
221666_s_at	-5.229	1.779E-04	PYCARD	PYD and CARD domain containing	proteolysis /// induction of apoptosis /// caspase activation /// cell cycle /// signal transduction /// regulation of apoptosis /// negative regulation of progression through cell cycle /// positive regulation of interleukin-1 beta secretion /// positive regulation of caspase activity /// apoptosis	protein binding /// caspase activator activity /// caspase activity /// Pyrin domain binding /// protein homodimerization activity /// protein binding	intracellular /// cytoplasm /// cytoplasm	---
202207_at	-6.256	4.215E-05	ARL4C	ADP-ribosylation factor-like 4C	rRNA processing /// small GTPase mediated signal transduction /// ribosome biogenesis	nucleotide binding /// GTPase activity /// GTP binding	intracellular /// nucleus /// nucleus	---
213139_at	-7.666	7.060E-06	SNAI2	snail homolog 2 (Drosophila)	negative regulation of transcription from RNA polymerase II promoter /// transcription /// regulation of transcription, DNA-dependent /// development /// ectoderm and mesoderm interaction /// sensory perception of sound	DNA binding /// zinc ion binding /// metal ion binding /// nucleic acid binding	intracellular /// nucleus /// nucleus	---
205573_s_at	-5.573	1.088E-04	SNX7	sorting nexin 7	intracellular signaling cascade /// protein transport /// transport	protein binding /// phosphoinositide binding	---	---

221016_s_at	-7.337	1.023E-05	TCF7L1	transcription factor 7-like 1 (T-cell specific, HMG-box) /// transcription factor 7-like 1 (T-cell specific, HMG-box)	establishment and/or maintenance of chromatin architecture /// transcription /// regulation of transcription, DNA-dependent /// regulation of Wnt receptor signaling pathway /// regulation of transcription, DNA-dependent /// Wnt receptor signaling pathway	transcription factor activity /// DNA binding /// DNA binding	nucleus /// nucleus	---
201336_at	-5.359	1.492E-04	VAMP3	vesicle-associated membrane protein 3 (cellubrevin)	protein complex assembly /// vesicle docking during exocytosis /// membrane fusion /// vesicle-mediated transport /// exocytosis	protein binding	membrane /// integral to membrane /// synaptosome /// synapse /// integral to membrane	---
201300_s_at	-5.341	1.518E-04	PRNP	prion protein (p27-30) (Creutzfeldt-Jakob disease, Gerstmann-Strausler-Scheinker syndrome, fatal familial insomnia)	copper ion homeostasis /// response to oxidative stress /// metabolism	copper ion binding /// microtubule binding /// GPI anchor binding /// tubulin binding	cytoplasm /// endoplasmic reticulum /// Golgi apparatus /// plasma membrane /// extrinsic to membrane /// lipid raft /// membrane	---
221485_at	-5.069	2.351E-04	B4GALT5	UDP-Gal:betaGlcNAc beta 1,4-galactosyltransferase, polypeptide 5	carbohydrate metabolism	galactosyltransferase activity /// transferase activity, transferring glycosyl groups /// manganese ion binding /// metal ion binding /// transferase activity	membrane /// integral to membrane	N-Glycan biosynthesis /// Keratan sulfate biosynthesis /// Glycan structures - biosynthesis 1
211564_s_at	-5.197	1.884E-04	PDLIM4	PDZ and LIM domain 4	---	protein binding /// zinc ion binding /// metal ion binding /// protein binding	---	---
212724_at	-5.95	5.829E-05	RND3	Rho family GTPase 3	small GTPase mediated signal transduction	nucleotide binding /// GTP binding	intracellular /// membrane	---
201560_at	-5.455	1.284E-04	CLIC4	chloride intracellular channel 4	ion transport /// chloride transport /// cell differentiation /// negative regulation of cell migration /// transport /// chloride transport	voltage-gated chloride channel activity /// protein binding /// chloride ion binding /// ion channel activity /// voltage-gated ion channel activity /// chloride channel activity /// chloride channel activity	soluble fraction /// cytoplasm /// microvillus /// actin cytoskeleton /// membrane	---
202073_at	-5.254	1.719E-04	OPTN	optineurin	---	---	---	---
200609_s_at	-6.29	4.007E-05	WDR1	WD repeat domain 1	sensory perception of sound	actin binding /// protein binding	cytoskeleton	Hypertrophy_model /// Hypertrophy_model

202440_s_at	-6.733	2.223E-05	ST5	suppression of tumorigenicity 5	---	---	---	Ribosomal_Proteins
204344_s_at	-6.597	2.558E-05	SEC23A	Sec23 homolog A (S. cerevisiae)	intracellular protein transport /// ER to Golgi vesicle-mediated transport /// vesicle-mediated transport /// transport /// protein transport	protein binding /// zinc ion binding /// protein binding	endoplasmic reticulum /// cytosol /// COPII vesicle coat /// endoplasmic reticulum	---
203340_s_at	-5.811	7.415E-05	SLC25A12	solute carrier family 25 (mitochondrial carrier, Aralar), member 12	transport /// transport	transporter activity /// binding /// calcium ion binding /// solute:cation symporter activity	mitochondrion /// mitochondrial inner membrane /// integral to plasma membrane /// membrane /// mitochondrion /// integral to membrane	---
216215_s_at	-5.356	1.492E-04	RPL41	Ribosomal protein L41	protein biosynthesis /// negative regulation of transcription /// estrogen receptor signaling pathway /// RNA metabolism /// regulation of cell proliferation	structural constituent of ribosome /// protein binding /// transcription corepressor activity /// RNA binding /// transcription factor binding /// nucleotide binding /// nucleic acid binding /// RNA binding	ribosome /// nucleus /// ribonucleoprotein complex	Ribosomal_Proteins /// Ribosomal_Proteins /// Ribosomal_Proteins
214104_at	-6.215	4.242E-05	GPR161	G protein-coupled receptor 161	signal transduction /// G-protein coupled receptor protein signaling pathway	rhodopsin-like receptor activity /// receptor activity /// signal transducer activity /// G-protein coupled receptor activity	membrane /// integral to membrane	GPCRDB_Class_A_Rhodopsin-like2
212647_at	-5.181	1.934E-04	RRAS	related RAS viral (r-ras) oncogene homolog	Ras protein signal transduction /// small GTPase mediated signal transduction	nucleotide binding /// GTPase activity /// GTP binding	intracellular /// membrane	G_Protein_Signaling /// MAPK_Cascade
205803_s_at	-5.618	1.005E-04	TRPC1	transient receptor potential cation channel, subfamily C, member 1	ion transport /// calcium ion transport /// transport /// calcium ion transport	ion channel activity /// calcium ion binding /// store-operated calcium channel activity /// calcium channel activity /// calcium channel activity	plasma membrane /// integral to plasma membrane /// membrane /// integral to membrane	---
218310_at	-6.818	2.065E-05	RABGEF1	RAB guanine nucleotide exchange factor (GEF) 1	endocytosis /// protein transport /// transport	DNA binding /// protein binding /// zinc ion binding /// metal ion binding	---	---
220230_s_at	-5.653	9.755E-05	CYB5R2	cytochrome b5 reductase 2	electron transport /// electron transport	cytochrome-b5 reductase activity /// oxidoreductase activity /// FAD binding /// NAD binding /// cytochrome-b5 reductase activity	soluble fraction /// membrane	---

203423_at	-6.162	4.465E-05	RBP1	retinol binding protein 1, cellular	vitamin A metabolism /// transport	lipid binding /// retinal binding /// retinol binding /// binding /// retinoid binding	---	---
222240_s_at	-5.919	6.118E-05	ISYNA1	myo-inositol 1-phosphate synthase A1	myo-inositol biosynthesis /// phospholipid biosynthesis /// protein biosynthesis /// protein biosynthesis	inositol-3-phosphate synthase activity /// structural constituent of ribosome /// nucleic acid binding /// structural constituent of ribosome /// isomerase activity /// RNA binding /// structural constituent of ribosome	cytosolic small ribosomal subunit (sensu Eukaryota) /// intracellular /// ribosome /// small ribosomal subunit /// ribonucleoprotein complex /// cytosolic small ribosomal subunit (sensu Eukaryota)	Ribosomal_Proteins
219936_s_at	-9.397	1.317E-06	GPR87	G protein-coupled receptor 87	signal transduction /// G-protein coupled receptor protein signaling pathway	rhodopsin-like receptor activity /// receptor activity /// G-protein coupled receptor activity, unknown ligand /// purinergic nucleotide receptor activity, G-protein coupled /// signal transducer activity /// G-protein coupled receptor activity	membrane /// integral to membrane /// integral to membrane	GPCRDB_Class_A_Rhodopsin-like
204990_s_at	-6.131	4.761E-05	ITGB4	integrin, beta 4	cell communication /// cell adhesion /// cell-matrix adhesion /// integrin-mediated signaling pathway /// development /// cell adhesion	receptor activity /// protein binding /// protein binding	integrin complex /// membrane /// integral to membrane /// integrin complex	Integrin-mediated_cell_adhesion_KEGG
204636_at	-6.343	3.731E-05	COL17A1	collagen, type XVII, alpha 1	electron transport /// phosphate transport /// cell-matrix adhesion /// epidermis development	monooxygenase activity /// structural molecule activity /// iron ion binding /// heme binding	extracellular matrix (sensu Metazoa) /// cytoplasm /// microsome /// integral to plasma membrane /// intercellular junction /// membrane /// integral to membrane	---

203499_at	-6.886	1.797E-05	EPHA2	EPH receptor A2	protein amino acid phosphorylation /// signal transduction /// transmembrane receptor protein tyrosine kinase signaling pathway /// development	nucleotide binding /// protein-tyrosine kinase activity /// receptor activity /// ephrin receptor activity /// ATP binding /// transferase activity /// protein kinase activity /// kinase activity /// transmembrane receptor protein tyrosine kinase activity	integral to plasma membrane /// membrane /// integral to membrane	Calcium signaling pathway /// Cytokine-cytokine receptor interaction /// Apoptosis /// Dorso-ventral axis formation /// Axon guidance /// VEGF signaling pathway /// Focal adhesion /// Adherens junction /// Gap junction /// Hematopoietic cell lineage /// Long-term depression /// Regulation of actin cytoskeleton /// Insulin signaling pathway /// GnRH signaling pathway /// Type II diabetes mellitus /// Dentatorubropallidoluysian atrophy (DRPLA) /// Epithelial cell signaling in Helicobacter pylori /// Colorectal cancer
209270_at	-6.635	2.510E-05	LAMB3	laminin, beta 3	cell adhesion /// epidermis development	structural molecule activity /// protein binding	extracellular matrix (sensu Metazoa) /// basement membrane /// laminin-5	---
209016_s_at	-6.23	4.242E-05	KRT7	keratin 7	cytoskeleton organization and biogenesis	structural molecule activity /// protein binding /// identical protein binding /// structural molecule activity /// zinc ion binding /// metal ion binding	intermediate filament /// intermediate filament /// intracellular	---

205157_s_at	-5.207	1.850E-04	KRT17	keratin 17	epidermis development /// sarcomere organization /// cell proliferation /// cytoskeleton organization and biogenesis	structural molecule activity /// structural constituent of cytoskeleton /// protein binding /// structural constituent of muscle /// structural constituent of cytoskeleton /// structural constituent of epidermis /// structural constituent of cytoskeleton	intermediate filament /// intermediate filament /// costamere /// intermediate filament /// intermediate filament	---
209863_s_at	-6.889	1.797E-05	TP73L	tumor protein p73-like	transcription /// apoptosis /// induction of apoptosis /// Notch signaling pathway /// development /// positive regulation of Notch signaling pathway /// negative regulation of transcription, DNA-dependent /// positive regulation of transcription, DNA-dependent /// protein homotetramerization /// regulation of transcription, DNA-dependent /// apoptosis	transcription factor activity /// zinc ion binding /// transcriptional activator activity /// transcriptional repressor activity /// identical protein binding /// metal ion binding /// protein binding /// DNA binding /// DNA binding	nucleus /// nucleus	---
201820_at	-6.438	3.219E-05	KRT5	keratin 5 (epidermolysis bullosa simplex, Dowling-Meara/Kobner/Weber-Cockayne types)	epidermis development /// ectoderm development /// cytoskeleton organization and biogenesis	structural molecule activity /// structural constituent of cytoskeleton /// protein binding /// protein binding /// structural molecule activity /// structural constituent of cytoskeleton	intermediate filament /// intermediate filament /// intermediate filament /// intermediate filament	---

204455_at	-7.744	6.745E-06	DST	dystonin	cytoskeleton organization and biogenesis /// cell cycle arrest /// cell adhesion /// integrin-mediated signaling pathway /// actin cytoskeleton organization and biogenesis /// intermediate filament cytoskeleton organization and biogenesis /// intermediate filament cytoskeleton organization and biogenesis /// intermediate filament cytoskeleton organization and biogenesis	actin binding /// integrin binding /// structural constituent of cytoskeleton /// structural constituent of cytoskeleton /// calcium ion binding /// protein binding /// protein C-terminus binding /// actin filament binding /// protein binding /// structural molecule activity	basement membrane /// extracellular space /// cytoplasm /// cytoplasm /// cytoskeleton /// intercellular junction /// cytoplasmic membrane-bound vesicle /// hemidesmosome /// basal plasma membrane	---
204971_at	-9.11	1.317E-06	CSTA	cystatin A (stefin A)	peptide cross-linking /// keratinocyte differentiation	cysteine protease inhibitor activity /// structural molecule activity /// protein binding, bridging /// endopeptidase inhibitor activity /// endopeptidase inhibitor activity /// cysteine protease inhibitor activity	cornified envelope /// intracellular	---
209351_at	-6.025	5.602E-05	KRT14	keratin 14 (epidermolysis bullosa simplex, Dowling-Meara, Koebner)	epidermis development	structural constituent of cytoskeleton /// protein binding /// structural constituent of epidermis /// structural molecule activity	intermediate filament /// intermediate filament	---
219024_at	-5.055	2.372E-04	PLEKHA1	pleckstrin homology domain containing, family A (phosphoinositide binding specific) member 1	---	phospholipid binding /// lipid binding	nucleus /// membrane	---
202504_at	-6.089	5.084E-05	TRIM29	tripartite motif-containing 29	transcription from RNA polymerase II promoter	transcription factor activity /// protein binding /// zinc ion binding /// metal ion binding	intracellular	---

31845_at	-6.004	5.675E-05	ELF4	E74-like factor 4 (ets domain transcription factor)	natural killer cell proliferation /// NK T cell proliferation /// transcription /// regulation of transcription, DNA-dependent /// positive regulation of transcription from RNA polymerase II promoter	transcription factor activity /// protein binding /// transcriptional activator activity /// sequence-specific DNA binding /// DNA binding	nucleus /// PML body	---
205543_at	-6.911	1.797E-05	HSPA4L	heat shock 70kDa protein 4-like	protein folding /// response to unfolded protein /// protein folding /// response to unfolded protein /// response to unfolded protein	nucleotide binding /// ATP binding /// unfolded protein binding /// ATP binding	nucleus /// cytoplasm /// nucleus	---
201850_at	-5.851	6.877E-05	CAPG	capping protein (actin filament), gelsolin-like	protein complex assembly /// response to pest, pathogen or parasite /// barbed-end actin filament capping /// barbed-end actin filament capping	actin binding	nucleus /// F-actin capping protein complex	---
201605_x_at	-5.429	1.346E-04	CNN2	calponin 2	cytoskeleton organization and biogenesis /// actomyosin structure organization and biogenesis	actin binding /// calmodulin binding /// actin binding	cytoskeleton /// intercellular junction	Smooth_muscle_contraction
212373_at	-5.066	2.351E-04	FEM1B	fem-1 homolog b (C. elegans)	induction of apoptosis	receptor activity /// death receptor binding	---	---
205018_s_at	-5.032	2.458E-04	MBNL2	muscleblind-like 2 (Drosophila)	---	nucleic acid binding /// zinc ion binding	---	---
219250_s_at	-5.461	1.279E-04	FLRT3	fibronectin leucine rich transmembrane protein 3	cell adhesion	receptor signaling protein activity /// protein binding, bridging /// protein binding	extracellular matrix (sensu Metazoa) /// integral to plasma membrane /// membrane /// integral to membrane	---
204734_at	-5.291	1.636E-04	KRT15	keratin 15	epidermis development	structural constituent of cytoskeleton /// protein binding /// structural molecule activity	intermediate filament /// intermediate filament	---
203780_at	-5.241	1.758E-04	EVA1	epithelial V-like antigen 1	cell adhesion /// homophilic cell adhesion /// morphogenesis	protein binding	cytoskeleton /// membrane /// integral to membrane	---

205044_at	-6.22	4.242E-05	GABRP	gamma-aminobutyric acid (GABA) A receptor, pi	ion transport /// transport	GABA-A receptor activity /// ion channel activity /// extracellular ligand-gated ion channel activity /// chloride channel activity /// neurotransmitter receptor activity /// chloride ion binding /// GABA-A receptor activity /// receptor activity	integral to membrane /// postsynaptic membrane /// membrane	---
210096_at	-6.394	3.469E-05	CYP4B1	cytochrome P450, family 4, subfamily B, polypeptide 1	electron transport	monooxygenase activity /// iron ion binding /// oxygen binding /// heme binding /// metal ion binding /// unspecific monooxygenase activity /// oxidoreductase activity	endoplasmic reticulum /// microsome /// membrane	Fatty acid metabolism /// gamma-Hexachlorocyclohexane degradation /// Tryptophan metabolism /// Arachidonic acid metabolism /// Linoleic acid metabolism /// Metabolism of xenobiotics by cytochrome P450

208228_s_at	-6.443	3.219E-05	FGFR2	fibroblast growth factor receptor 2 (bacteria-expressed kinase, keratinocyte growth factor receptor, craniofacial dysostosis 1, Crouzon syndrome, Pfeiffer syndrome, Jackson-Weiss syndrome)	protein amino acid phosphorylation /// protein amino acid phosphorylation /// cell growth	nucleotide binding /// protein-tyrosine kinase activity /// protein-tyrosine kinase activity /// receptor activity /// fibroblast growth factor receptor activity /// ATP binding /// heparin binding /// transferase activity /// protein kinase activity /// kinase activity	membrane /// membrane /// integral to membrane /// integral to membrane	Calcium signaling pathway /// Cytokine-cytokine receptor interaction /// Apoptosis /// Dorso-ventral axis formation /// Axon guidance /// VEGF signaling pathway /// Focal adhesion /// Adherens junction /// Gap junction /// Hematopoietic cell lineage /// Long-term depression /// Regulation of actin cytoskeleton /// Insulin signaling pathway /// GnRH signaling pathway /// Type II diabetes mellitus /// Dentatorubropallidoluysian atrophy (DRPLA) /// Epithelial cell signaling in Helicobacter pylori /// Colorectal cancer
207291_at	-5.474	1.247E-04	PRRG4	proline rich Gla (G-carboxyglutamic acid) 4 (transmembrane)	---	calcium ion binding	extracellular region /// membrane /// integral to membrane /// integral to membrane	---
205945_at	-5.31	1.606E-04	IL6R	interleukin 6 receptor /// interleukin 6 receptor	immune response /// cell surface receptor linked signal transduction /// development /// cell proliferation	receptor activity /// hematopoietin/interferon-class (D200-domain) cytokine receptor activity /// interleukin-6 receptor activity /// enzyme binding /// protein binding	extracellular region /// interleukin-6 receptor complex /// membrane /// integral to membrane /// integral to plasma membrane	---
211340_s_at	-6.423	3.283E-05	MCAM	melanoma cell adhesion molecule	cell adhesion /// morphogenesis /// cell adhesion	protein binding	plasma membrane /// integral to plasma membrane /// membrane /// integral to membrane	---

204149_s_at	-5.67	9.484E-05	GSTM4	glutathione S-transferase M4	metabolism	glutathione transferase activity /// transferase activity /// glutathione transferase activity	---	Glutathione metabolism /// Metabolism of xenobiotics by cytochrome P450
205578_at	-6.071	5.213E-05	ROR2	receptor tyrosine kinase-like orphan receptor 2	protein amino acid phosphorylation /// signal transduction /// development /// development	nucleotide binding /// transmembrane receptor protein tyrosine kinase activity /// receptor activity /// ATP binding /// transferase activity /// protein kinase activity /// protein-tyrosine kinase activity /// kinase activity	integral to plasma membrane /// membrane /// integral to membrane	---
204735_at	-9.041	1.317E-06	PDE4A	phosphodiesterase 4A, cAMP-specific (phosphodiesterase E2 dunce homolog, Drosophila)	signal transduction /// signal transduction /// cyclic nucleotide metabolism	catalytic activity /// 3',5'-cyclic-nucleotide phosphodiesterase activity /// 3',5'-cyclic-AMP phosphodiesterase activity /// 3',5'-cyclic-AMP phosphodiesterase activity /// hydrolase activity	membrane fraction /// soluble fraction /// insoluble fraction	G_Protein_Signaling /// Purine metabolism
204457_s_at	-5.567	1.089E-04	GAS1	growth arrest-specific 1	cell cycle /// cell cycle arrest /// negative regulation of cell proliferation /// negative regulation of S phase of mitotic cell cycle /// cell cycle arrest	GPI anchor binding	membrane /// anchored to plasma membrane	---
201121_s_at	-7.246	1.180E-05	PGRMC1	progesterone receptor membrane component 1	---	receptor activity /// steroid binding /// lipid binding /// steroid binding	endoplasmic reticulum /// microsome /// integral to plasma membrane /// cell surface /// membrane /// integral to membrane	---
209821_at	-5.646	9.755E-05	C9orf26	chromosome 9 open reading frame 26 (NF-HEV)	---	---	nucleus	---
202401_s_at	-6.739	2.223E-05	SRF	serum response factor (c-fos serum response element-binding transcription factor)	transcription /// regulation of transcription from RNA polymerase II promoter /// signal transduction /// development /// regulation of transcription, DNA-dependent	transcription factor activity /// RNA polymerase II transcription factor activity /// transcription factor binding /// sequence-specific DNA binding /// protein binding /// DNA binding	nucleus /// nucleus	---

202133_at	-5.953	5.829E-05	WWTR1	WW domain containing transcription regulator 1	transcription /// regulation of transcription, DNA-dependent /// regulation of transcription, DNA-dependent	transcription coactivator activity	nucleus /// nucleus	---
202724_s_at	-5.438	1.320E-04	FOXO1A	forkhead box O1A (rhabdomyosarcoma)	transcription /// regulation of transcription, DNA-dependent /// regulation of transcription from RNA polymerase II promoter /// anti-apoptosis	transcription factor activity /// transcription factor activity /// sequence-specific DNA binding /// DNA binding	nucleus	---
37996_s_at	-5.596	1.051E-04	DMPK	dystrophia myotonica-protein kinase	protein amino acid phosphorylation /// protein amino acid phosphorylation /// muscle contraction /// regulation of heart contraction /// regulation of small GTPase mediated signal transduction /// protein modification	nucleotide binding /// magnesium ion binding /// protein serine/threonine kinase activity /// protein serine/threonine kinase activity /// ATP binding /// ATP binding /// transferase activity /// identical protein binding /// protein binding /// protein kinase activity /// kinase activity /// metal ion binding /// protein kinase activity	---	---
219935_at	-5.034	2.458E-04	ADAMTS5	ADAM metallopeptidase with thrombospondin type 1 motif, 5 (aggrecanase-2)	proteolysis /// proteolysis	metalloendopeptidase activity /// integrin binding /// zinc ion binding /// metal ion binding /// peptidase activity /// metallopeptidase activity /// hydrolase activity /// metallopeptidase activity	extracellular matrix (sensu Metazoa) /// extracellular matrix /// extracellular matrix (sensu Metazoa)	---
201603_at	-5.853	6.877E-05	PPP1R12A	protein phosphatase 1, regulatory (inhibitor) subunit 12A	regulation of muscle contraction	signal transducer activity	actin cytoskeleton	---
207053_at	-6.489	3.019E-05	SLC8A1	solute carrier family 8 (sodium/calcium exchanger), member 1	ion transport /// sodium ion transport /// calcium ion transport /// calcium ion transport /// muscle contraction /// cell communication /// transport /// sodium ion transport	calcium:sodium antiporter activity /// calcium ion binding /// calmodulin binding /// sodium ion transporter activity /// calcium ion transporter activity /// antiporter activity /// heat shock protein binding /// sodium ion binding	integral to plasma membrane /// membrane /// integral to membrane	Calcium_regulation_in_cardiac_cells /// Smooth_muscle_contraction

204731_at	-5.953	5.829E-05	TGFBR3	transforming growth factor, beta receptor III (betaglycan, 300kDa)	signal transduction /// transforming growth factor beta receptor signaling pathway /// development	receptor activity /// glycosaminoglycan binding /// receptor activity	membrane /// integral to membrane /// integral to membrane	TGF_Beta_Signaling_Pathway
219647_at	-5.781	7.767E-05	POPDC2	popeye domain containing 2	---	---	membrane /// integral to membrane /// integral to membrane	Electron_Transport_Chain
216733_s_at	-5.641	9.755E-05	GATM	glycine amidinotransferase (L-arginine:glycine amidinotransferase)	creatine biosynthesis	glycine amidinotransferase activity /// transferase activity /// glycine amidinotransferase activity	mitochondrion /// mitochondrial intermembrane space	Urea cycle and metabolism of amino groups /// Glycine, serine and threonine metabolism /// Arginine and proline metabolism

211985_s_at	-5.276	1.668E-04	CALM1	calmodulin 1 (phosphorylase kinase, delta)	G-protein coupled receptor protein signaling pathway /// G-protein coupled receptor protein signaling pathway	protein binding /// N-terminal myristoylation domain binding /// calcium ion binding /// protein binding /// calcium ion binding /// calcium ion binding /// protein binding /// protein binding	cytoplasm /// plasma membrane /// cytoplasm /// plasma membrane	G_Protein_Signaling /// G13_Signaling_Pathway /// Glycogen_Metabolism /// Calcium_regulation_in_cardiac_cells /// Smooth_muscle_contraction /// G_Protein_Signaling /// G13_Signaling_Pathway /// Calcium_regulation_in_cardiac_cells /// Smooth_muscle_contraction /// Calcium_regulation_in_cardiac_cells /// Smooth_muscle_contraction /// G_Protein_Signaling /// G13_Signaling_Pathway /// G_Protein_Signaling /// G13_Signaling_Pathway /// Glycogen_Metabolism /// Calcium_regulation_in_cardiac_cells /// Smooth_muscle_contraction
209283_at	-7.811	6.745E-06	CRYAB	crystallin, alpha B	protein folding /// muscle contraction /// transmembrane receptor protein tyrosine kinase signaling pathway /// visual perception	structural constituent of eye lens /// unfolded protein binding /// protein binding	nucleus /// cytoplasm	---

203632_s_at	-5.629	9.892E-05	GPRC5B	G protein-coupled receptor, family C, group 5, member B	signal transduction /// G-protein coupled receptor protein signaling pathway /// visual perception	receptor activity /// sevenless binding /// metabotropic glutamate, GABA-B-like receptor activity /// signal transducer activity /// G-protein coupled receptor activity	membrane /// integral to membrane	GPCRDB_Class_C_Metabotropic_glutamate_pheromone
203296_s_at	-5.37	1.471E-04	ATP1A2	ATPase, Na+/K+ transporting, alpha 2 (+) polypeptide	potassium ion transport /// sodium ion transport /// metabolism /// monovalent inorganic cation transport /// ATP hydrolysis coupled proton transport /// sperm motility /// hydrogen ion homeostasis /// transport /// ion transport /// cation transport /// potassium ion transport /// sodium ion transport	nucleotide binding /// magnesium ion binding /// sodium:potassium-exchanging ATPase activity /// ATP binding /// monovalent inorganic cation transporter activity /// ATPase activity, coupled to transmembrane movement of ions, phosphorylative mechanism /// hydrolase activity /// hydrolase activity, acting on acid anhydrides, catalyzing transmembrane movement of substances /// potassium ion binding /// sodium ion binding /// catalytic activity /// sodium:potassium-exchanging ATPase activity /// metal ion binding /// sodium:potassium-exchanging ATPase activity	sodium:potassium-exchanging ATPase complex /// membrane /// integral to membrane /// integral to plasma membrane /// sodium:potassium-exchanging ATPase complex	---
219737_s_at	-5.285	1.645E-04	PCDH9	protocadherin 9	cell adhesion /// homophilic cell adhesion	calcium ion binding /// protein binding	membrane /// integral to membrane	---
206938_at	-5.582	1.081E-04	SRD5A2	steroid-5-alpha-reductase, alpha polypeptide 2 (3-oxo-5 alpha-steroid delta 4-dehydrogenase alpha 2)	cell-cell signaling /// sex determination /// sex differentiation /// androgen metabolism /// male gonad development /// cell differentiation	3-oxo-5-alpha-steroid 4-dehydrogenase activity /// sterol 5-alpha reductase activity /// oxidoreductase activity	endoplasmic reticulum /// microsome /// membrane /// integral to membrane	Bile acid biosynthesis /// Androgen and estrogen metabolism
209747_at	-5.236	1.772E-04	TGFB3	transforming growth factor, beta 3	regulation of progression through cell cycle /// signal transduction /// cell-cell signaling /// cell proliferation /// organ morphogenesis /// cell growth /// growth	transforming growth factor beta receptor binding /// growth factor activity /// transforming growth factor beta receptor binding	---	---

211343_s_at	-7.696	7.025E-06	COL13A1	collagen, type XIII, alpha 1	phosphate transport	---	collagen type XIII /// cytoplasm /// plasma membrane	---
202920_at	-5.754	8.283E-05	ANK2	ankyrin 2, neuronal	signal transduction	structural constituent of cytoskeleton /// protein binding	actin cytoskeleton /// membrane /// cytoskeleton	---
205382_s_at	-6.213	4.242E-05	CFD	complement factor D (adipsin)	proteolysis /// proteolysis /// complement activation, alternative pathway /// innate immune response /// caspase activation /// immune response /// protein folding /// response to unfolded protein /// complement activation /// caspase activation	complement factor D activity /// complement factor D activity /// peptidase activity /// chaperone binding /// unfolded protein binding /// chaperone binding /// serine-type endopeptidase activity /// hydrolase activity /// ATP binding /// unfolded protein binding /// serine-type peptidase activity /// unfolded protein binding	mitochondrion /// mitochondrion /// mitochondrion	Complement and coagulation cascades
204993_at	-5.527	1.160E-04	GNAZ	guanine nucleotide binding protein (G protein), alpha z polypeptide	signal transduction /// G-protein coupled receptor protein signaling pathway /// G-protein coupled receptor protein signaling pathway	nucleotide binding /// GTPase activity /// receptor signaling protein activity /// GTP binding /// signal transducer activity /// guanyl nucleotide binding	nuclear envelope /// endoplasmic reticulum /// plasma membrane /// membrane	G_Protein_Signaling /// G_Protein_Signaling /// G_Protein_Signaling /// Calcium_regulation_in_cardiac_cells /// Calcium_regulation_in_cardiac_cells /// Calcium_regulation_in_cardiac_cells
219167_at	-5.256	1.715E-04	RASL12	RAS-like, family 12	small GTPase mediated signal transduction	nucleotide binding /// GTP binding	membrane	---
203903_s_at	-5.02	2.485E-04	HEPH	hephaestin	ion transport /// copper ion transport /// iron ion transport /// transport	copper ion transporter activity /// iron ion binding /// copper ion binding /// oxidoreductase activity /// metal ion binding	membrane /// integral to membrane	---

211535_s_at	-7.062	1.472E-05	FGFR1	fibroblast growth factor receptor 1 (fms-related tyrosine kinase 2, Pfeiffer syndrome)	MAPKKK cascade /// skeletal development /// protein amino acid phosphorylation /// fibroblast growth factor receptor signaling pathway /// cell growth /// protein amino acid phosphorylation /// fibroblast growth factor receptor signaling pathway	nucleotide binding /// protein-tyrosine kinase activity /// receptor activity /// fibroblast growth factor receptor activity /// fibroblast growth factor receptor activity /// protein binding /// ATP binding /// heparin binding /// transferase activity /// protein kinase activity /// protein-tyrosine kinase activity /// ATP binding /// heparin binding /// kinase activity	membrane fraction /// integral to plasma membrane /// membrane /// integral to membrane /// integral to membrane	Calcium signaling pathway /// Cytokine-cytokine receptor interaction /// Apoptosis /// Dorso-ventral axis formation /// Axon guidance /// VEGF signaling pathway /// Focal adhesion /// Adherens junction /// Gap junction /// Hematopoietic cell lineage /// Long-term depression /// Regulation of actin cytoskeleton /// Insulin signaling pathway /// GnRH signaling pathway /// Type II diabetes mellitus /// Dentatorubropallidoluysian atrophy (DRPLA) /// Epithelial cell signaling in Helicobacter pylori /// Colorectal cancer
219902_at	-7.765	6.745E-06	BHMT2	betaine-homocysteine methyltransferase 2	---	methyltransferase activity /// homocysteine S-methyltransferase activity /// transferase activity	---	---
205475_at	-5.717	8.796E-05	SCRG1	scrapie responsive protein 1	nervous system development	---	extracellular space	---
207876_s_at	-7.21	1.180E-05	FLNC	filamin C, gamma (actin binding protein 280)	---	actin binding /// protein binding /// actin binding	actin cytoskeleton /// cytoskeleton	---

205132_at	-8.113	4.812E-06	ACTC1	actin, alpha, cardiac muscle 1	---	nucleotide binding /// structural constituent of cytoskeleton /// protein binding /// ATP binding /// structural molecule activity	cytoskeleton /// actin filament	Smooth_muscle_contraction /// Striated_muscle_contraction /// Smooth_muscle_contraction /// Striated_muscle_contraction
204940_at	-8.084	4.812E-06	PLN	phospholamban	calcium ion transport /// muscle contraction /// circulation /// calcium ion transport	calcium channel regulator activity /// protein binding /// ATPase inhibitor activity /// calcium channel regulator activity	smooth endoplasmic reticulum /// membrane /// integral to membrane	Calcium_regulation_in_cardiac_cells
205304_s_at	-5.08	2.308E-04	KCNJ8	potassium inwardly-rectifying channel, subfamily J, member 8	ion transport /// potassium ion transport /// transport /// potassium ion transport	voltage-gated ion channel activity /// ATP-activated inward rectifier potassium channel activity /// potassium ion binding /// ion channel activity /// inward rectifier potassium channel activity /// inward rectifier potassium channel activity	membrane fraction /// voltage-gated potassium channel complex /// membrane /// integral to membrane	---
201234_at	-5.842	6.956E-05	ILK	integrin-linked kinase	protein amino acid phosphorylation /// cell-matrix adhesion /// integrin-mediated signaling pathway /// integrin-mediated signaling pathway /// protein amino acid phosphorylation /// integrin-mediated signaling pathway /// cell-matrix adhesion /// integrin-mediated signaling pathway /// cell proliferation	nucleotide binding /// protein serine/threonine kinase activity /// protein-tyrosine kinase activity /// ATP binding /// kinase activity /// transferase activity /// protein kinase activity /// protein serine/threonine kinase activity /// protein serine/threonine kinase activity /// ATP binding	cytoplasm	Integrin-mediated_cell_adhesion_KEGG /// RNA_transcription_Reactome /// Integrin-mediated_cell_adhesion_KEGG /// RNA_transcription_Reactome
204753_s_at	-5.425	1.358E-04	HLF	hepatic leukemia factor	transcription /// regulation of transcription, DNA-dependent /// transcription from RNA polymerase II promoter /// development /// rhythmic process	double-stranded DNA binding /// transcription factor activity /// sequence-specific DNA binding /// protein dimerization activity /// DNA binding /// DNA binding	nucleus /// nucleus	---

209616_s_at	-6.309	3.885E-05	CES1	carboxylesterase 1 (monocyte/macrophage serine esterase 1)	metabolism /// response to toxin	carboxylesterase activity /// serine esterase activity /// serine esterase activity /// hydrolase activity /// carboxylesterase activity	extracellular space /// endoplasmic reticulum	Irinotecan_pathway_PharmGKB /// Alkaloid biosynthesis II
209496_at	-5.578	1.083E-04	RARRES2	retinoic acid receptor responder (tazarotene induced) 2	retinoid metabolism	---	---	---
204163_at	-6.033	5.529E-05	EMILIN1	elastin microfibril interfacer 1	phosphate transport /// cell adhesion /// cell adhesion	extracellular matrix structural constituent /// protein binding /// protein binding	extracellular matrix (sensu Metazoa) /// cytoplasm /// extracellular matrix (sensu Metazoa)	---
204099_at	-6.693	2.304E-05	SMARCD3	SWI/SNF related, matrix associated, actin dependent regulator of chromatin, subfamily d, member 3	chromatin remodeling /// transcription /// regulation of transcription from RNA polymerase II promoter /// positive regulation of transcription, DNA-dependent /// regulation of transcription, DNA-dependent /// chromatin modification	transcription coactivator activity /// receptor binding /// transcription factor binding /// transcription regulator activity /// nuclear hormone receptor binding	nucleus /// nucleoplasm /// SWI/SNF complex /// nucleus	---
202222_s_at	-5.481	1.230E-04	DES	desmin	muscle contraction /// cytoskeleton organization and biogenesis /// regulation of heart contraction	structural molecule activity /// structural constituent of cytoskeleton /// protein binding	intermediate filament /// intermediate filament	Striated_muscle_contraction /// Striated_muscle_contraction
201957_at	-6.196	4.271E-05	PPP1R12B	protein phosphatase 1, regulatory (inhibitor) subunit 12B	regulation of muscle contraction /// signal transduction	enzyme activator activity	---	---
201496_x_at	-5.356	1.492E-04	MYH11	myosin, heavy polypeptide 11, smooth muscle	striated muscle contraction	nucleotide binding /// motor activity /// actin binding /// calmodulin binding /// ATP binding	muscle myosin /// striated muscle thick filament /// myosin	---
209948_at	-6.906	1.797E-05	KCNMB1	potassium large conductance calcium-activated channel, subfamily M, beta member 1	ion transport /// potassium ion transport /// smooth muscle contraction /// synaptic transmission /// transport /// potassium ion transport	ion channel activity /// calcium-activated potassium channel activity /// potassium channel regulator activity /// calcium-activated potassium channel activity	membrane /// integral to membrane	---

204041_at	-5.948	5.829E-05	MAOB	monoamine oxidase B	electron transport	amine oxidase activity /// electron carrier activity /// oxidoreductase activity	mitochondrion /// mitochondrial envelope /// membrane /// integral to membrane	Glycine, serine and threonine metabolism /// Arginine and proline metabolism /// Histidine metabolism /// Tyrosine metabolism /// Phenylalanine metabolism /// Tryptophan metabolism
203706_s_at	-5.082	2.308E-04	FZD7	frizzled homolog 7 (Drosophila)	G-protein coupled receptor protein signaling pathway /// frizzled signaling pathway /// development /// signal transduction /// cell surface receptor linked signal transduction /// Wnt receptor signaling pathway	receptor activity /// non-G-protein coupled 7TM receptor activity /// G-protein coupled receptor activity /// Wnt receptor activity /// signal transducer activity	plasma membrane /// integral to membrane /// membrane /// integral to membrane	Wnt_signaling
203951_at	-5.308	1.606E-04	CNN1	calponin 1, basic, smooth muscle	regulation of smooth muscle contraction /// actomyosin structure organization and biogenesis	actin binding /// calmodulin binding	---	Smooth_muscle_contraction
201431_s_at	-6.078	5.187E-05	DPYSL3	dihydropyrimidinase-like 3	nucleobase, nucleoside, nucleotide and nucleic acid metabolism /// signal transduction /// nervous system development	dihydropyrimidinase activity /// hydrolase activity	---	---
209199_s_at	-5.144	2.064E-04	MEF2C	MADS box transcription enhancer factor 2, polypeptide C (myocyte enhancer factor 2C)	transcription /// regulation of transcription, DNA-dependent /// transcription from RNA polymerase II promoter /// nervous system development /// muscle development	transcription factor activity /// RNA polymerase II transcription factor activity /// transcription coactivator activity /// sequence-specific DNA binding /// DNA binding /// transcription factor activity	nucleus	---
208131_s_at	-7.138	1.310E-05	PTGIS	prostaglandin I2 (prostacyclin) synthase /// prostaglandin I2 (prostacyclin) synthase	prostaglandin biosynthesis /// electron transport /// lipid metabolism /// fatty acid biosynthesis /// lipid biosynthesis /// prostaglandin metabolism	monooxygenase activity /// iron ion binding /// prostaglandin-I synthase activity /// isomerase activity /// heme binding /// metal ion binding /// prostaglandin-I synthase activity /// oxidoreductase activity	endoplasmic reticulum /// membrane /// integral to membrane /// microsome	Eicosanoid_Synthesis /// Prostaglandin_synthesis_regulation /// Arachidonic acid metabolism

205935_at	-7.48	8.324E-06	FOXF1	forkhead box F1	transcription /// regulation of transcription from RNA polymerase II promoter /// regulation of transcription, DNA-dependent /// transcription from RNA polymerase II promoter	transcription factor activity /// transcription coactivator activity /// protein binding /// sequence-specific DNA binding /// DNA binding /// transcription factor activity	nucleus /// transcription factor complex /// nucleus	---
209074_s_at	-6.683	2.316E-05	FAM107A	family with sequence similarity 107, member A	regulation of cell growth /// regulation of cell growth	---	nucleus /// nucleus	---
206631_at	-7.375	9.706E-06	PTGER2	prostaglandin E receptor 2 (subtype EP2), 53kDa	signal transduction /// G-protein coupled receptor protein signaling pathway /// G-protein coupled receptor protein signaling pathway	rhodopsin-like receptor activity /// receptor activity /// prostaglandin E receptor activity /// thromboxane receptor activity /// signal transducer activity /// G-protein coupled receptor activity /// prostaglandin E receptor activity	integral to plasma membrane /// membrane /// integral to membrane	Ovarian_Infertility_Genes /// Prostaglandin_synthesis_regulation /// GPCRDB_Class_A_Rhodopsin-like /// Small_ligand_GPCRs

207016_s_at	-5.538	1.137E-04	ALDH1A2	aldehyde dehydrogenase 1 family, member A2	vitamin A metabolism /// metabolism	retinal dehydrogenase activity /// 3-chloroallyl aldehyde dehydrogenase activity /// electron carrier activity /// oxidoreductase activity /// catalytic activity	---	Glycolysis / Gluconeogenesis /// Ascorbate and aldarate metabolism /// Fatty acid metabolism /// Bile acid biosynthesis /// Valine, leucine and isoleucine degradation /// Lysine degradation /// Arginine and proline metabolism /// Histidine metabolism /// Tryptophan metabolism /// beta-Alanine metabolism /// Glycerolipid metabolism /// Pyruvate metabolism /// 1,2-Dichloroethane degradation /// Propanoate metabolism /// Butanoate metabolism /// Limonene and pinene degradation
200621_at	-5.079	2.308E-04	CSRP1	cysteine and glycine-rich protein 1	---	zinc ion binding /// zinc ion binding /// metal ion binding	nucleus	---

209897_s_at	-5.234	1.772E-04	SLIT2	slit homolog 2 (Drosophila)	ureteric bud development /// chemotaxis /// G-protein coupled receptor protein signaling pathway /// nervous system development /// mesoderm migration /// sensory perception of smell /// neuron recognition /// motor axon guidance /// glial cell migration /// cell differentiation /// positive regulation of axonogenesis /// induction of negative chemotaxis /// induction of negative chemotaxis /// development /// nervous system development /// axon guidance	follicle stimulating hormone receptor activity /// receptor binding /// calcium ion binding /// protein binding	extracellular region /// extracellular space /// integral to membrane	---
221667_s_at	-5.702	9.062E-05	HSPB8	heat shock 22kDa protein 8	protein folding /// response to unfolded protein	protein serine/threonine kinase activity /// kinase activity /// transferase activity /// identical protein binding /// unfolded protein binding /// protein binding /// protein serine/threonine kinase activity	---	---
205404_at	-6.129	4.761E-05	HSD11B1	hydroxysteroid (11-beta) dehydrogenase 1	lipid metabolism /// metabolism /// glucocorticoid metabolism /// steroid metabolism	11-beta-hydroxysteroid dehydrogenase activity /// oxidoreductase activity /// 11-beta-hydroxysteroid dehydrogenase activity	endoplasmic reticulum /// endoplasmic reticulum membrane /// microsome /// membrane /// integral to membrane	Glucocorticoid_Mineralocorticoid_Metabolism /// Prostaglandin_synthesis_regulation /// C21-Steroid hormone metabolism /// Androgen and estrogen metabolism

204271_s_at	-5.93	6.047E-05	EDNRB	endothelin receptor type B	negative regulation of adenylate cyclase activity /// G-protein signaling, coupled to IP3 second messenger (phospholipase C activating) /// nervous system development /// sensory perception of sound /// signal transduction /// G-protein coupled receptor protein signaling pathway /// cell surface receptor linked signal transduction	rhodopsin-like receptor activity /// receptor activity /// endothelin receptor activity /// signal transducer activity /// G-protein coupled receptor activity /// endothelin receptor activity	plasma membrane /// integral to plasma membrane /// membrane /// integral to membrane	Prostaglandin_synthesis_regulation /// GPCRDB_Class_A_Rhodopsin-like /// Peptide_GPCRs
214577_at	-6.439	3.219E-05	MAP1B	microtubule-associated protein 1B	---	structural molecule activity /// protein binding	microtubule /// microtubule associated complex	---
205782_at	-6.618	2.517E-05	FGF7	fibroblast growth factor 7 (keratinocyte growth factor)	regulation of progression through cell cycle /// signal transduction /// cell-cell signaling /// cell proliferation /// positive regulation of cell proliferation /// epidermis development /// response to wounding	growth factor activity /// growth factor activity	extracellular region	---
203603_s_at	-5.208	1.850E-04	ZFHX1B	zinc finger homeobox 1b	regulation of transcription, DNA-dependent /// nervous system development /// negative regulation of transcription /// transcription /// regulation of transcription	transcription factor activity /// zinc ion binding /// transcriptional repressor activity /// phosphatase regulator activity /// sequence-specific DNA binding /// SMAD binding /// metal ion binding /// nucleic acid binding /// DNA binding	intracellular /// nucleus /// nucleus	TGF_Beta_Signaling_Pathway /// TGF_Beta_Signaling_Pathway
208792_s_at	-7.511	8.324E-06	CLU	clusterin	lipid metabolism /// apoptosis /// complement activation, classical pathway /// cell death /// innate immune response /// immune response /// complement activation	---	extracellular space	---

205168_at	-6.17	4.441E-05	DDR2	discoidin domain receptor family, member 2	protein amino acid phosphorylation /// cell adhesion /// cell adhesion /// signal transduction /// transmembrane receptor protein tyrosine kinase signaling pathway	nucleotide binding /// protein-tyrosine kinase activity /// transmembrane receptor protein tyrosine kinase activity /// receptor activity /// ATP binding /// transferase activity /// protein kinase activity /// transmembrane receptor protein tyrosine kinase activity /// kinase activity	integral to plasma membrane /// membrane /// integral to membrane	Calcium signaling pathway /// Cytokine-cytokine receptor interaction /// Apoptosis /// Dorso-ventral axis formation /// Axon guidance /// VEGF signaling pathway /// Focal adhesion /// Adherens junction /// Gap junction /// Hematopoietic cell lineage /// Long-term depression /// Regulation of actin cytoskeleton /// Insulin signaling pathway /// GnRH signaling pathway /// Type II diabetes mellitus /// Dentatorubropallidoluysian atrophy (DRPLA) /// Epithelial cell signaling in Helicobacter pylori /// Colorectal cancer
209487_at	-5.501	1.202E-04	RBPMS	RNA binding protein with multiple splicing	RNA processing	nucleotide binding /// RNA binding /// protein binding /// nucleic acid binding /// RNA binding	---	---
202748_at	-5.11	2.217E-04	GBP2	guanylate binding protein 2, interferon-inducible /// guanylate binding protein 2, interferon-inducible	immune response /// immune response	nucleotide binding /// GTPase activity /// GTP binding /// GTP binding	membrane	---
206580_s_at	-5.36	1.492E-04	EFEMP2	EGF-containing fibulin-like extracellular matrix protein 2	homophilic cell adhesion /// blood coagulation	transmembrane receptor activity /// extracellular matrix structural constituent /// calcium ion binding /// protein binding	basement membrane /// membrane	---

205011_at	-5.38	1.454E-04	LOH11CR2A	loss of heterozygosity, 11, chromosomal region 2, gene A	cell cycle /// negative regulation of progression through cell cycle	---	---	---
213800_at	-5.7	9.062E-05	CFH	complement factor H	complement activation, alternative pathway /// innate immune response /// immune response /// complement activation	---	extracellular space	---
209082_s_at	-5.288	1.636E-04	COL18A1	collagen, type XVIII, alpha 1	phosphate transport /// cell adhesion /// visual perception /// negative regulation of cell proliferation /// organ morphogenesis	structural molecule activity /// extracellular matrix structural constituent /// protein binding /// zinc ion binding /// metal ion binding	collagen /// cytoplasm /// extracellular matrix /// extracellular matrix (sensu Metazoa)	---
203921_at	-5.157	2.021E-04	CHST2	carbohydrate (N-acetylglucosamine-6-O) sulfotransferase 2	carbohydrate metabolism /// N-acetylglucosamine metabolism /// sulfur metabolism /// inflammatory response	N-acetylglucosamine 6-O-sulfotransferase activity /// transferase activity /// sulfotransferase activity	Golgi trans face /// membrane /// integral to membrane /// intrinsic to Golgi membrane	---
204396_s_at	-6.511	2.957E-05	GRK5	G protein-coupled receptor kinase 5	protein amino acid phosphorylation /// signal transduction /// G-protein signaling, coupled to cAMP nucleotide second messenger /// regulation of G-protein coupled receptor protein signaling pathway	nucleotide binding /// G-protein coupled receptor kinase activity /// signal transducer activity /// protein kinase C binding /// ATP binding /// phospholipid binding /// transferase activity /// protein kinase activity /// protein serine/threonine kinase activity /// kinase activity	soluble fraction /// cytoplasm	Calcium_regulation_in_cardiac_cells /// Smooth_muscle_contraction
200931_s_at	-5.051	2.374E-04	VCL	vinculin	cell motility /// cell adhesion /// lamellipodium biogenesis /// negative regulation of cell migration /// apical junction assembly /// cell adhesion	actin binding /// structural molecule activity /// oxidoreductase activity /// alpha-catenin binding /// protein binding /// protein binding /// actin binding /// protein binding	intercellular junction /// adherens junction /// focal adhesion /// actin cytoskeleton /// cell-matrix junction /// costamere /// protein complex /// cytoskeleton /// cytoskeleton	Integrin-mediated_cell_adhesion_KEGG

213746_s_at	-6.79	2.065E-05	FLNA	filamin A, alpha (actin binding protein 280)	cell motility /// cell surface receptor linked signal transduction /// nervous system development /// actin cytoskeleton organization and biogenesis /// positive regulation of I-kappaB kinase/NF-kappaB cascade /// positive regulation of I-kappaB kinase/NF-kappaB cascade	actin binding /// signal transducer activity /// protein binding /// actin filament binding /// signal transducer activity	actin cytoskeleton /// cytoskeleton	---
212136_at	-6.807	2.065E-05	ATP2B4	ATPase, Ca++ transporting, plasma membrane 4	cation transport /// calcium ion transport /// metabolism /// protein folding /// cellular protein metabolism /// transport /// ion transport /// regulation of apoptosis /// transport /// protein folding /// response to unfolded protein /// protein import into mitochondrial matrix	nucleotide binding /// magnesium ion binding /// calcium-transporting ATPase activity /// calcium ion binding /// calmodulin binding /// ATP binding /// hydrolase activity /// hydrolase activity, acting on acid anhydrides, catalyzing transmembrane movement of substances /// chaperone binding /// protein binding /// unfolded protein binding /// catalytic activity /// calcium-transporting ATPase activity /// calcium ion transporter activity /// ATPase activity, coupled to transmembrane movement of ions, phosphorylative mechanism /// metal ion binding /// unfolded protein binding /// ATP binding	plasma membrane /// integral to plasma membrane /// mitochondrion /// membrane /// integral to membrane /// cytoplasm /// mitochondrion	Calcium signaling pathway
202565_s_at	-6.898	1.797E-05	SVIL	supervillin	cytoskeleton organization and biogenesis /// striated muscle development /// barbed-end actin filament capping /// transport	calcium ion binding /// actin filament binding /// actin binding /// transporter activity	nucleus /// cytoplasm /// plasma membrane /// actin cytoskeleton /// costamere /// cytoskeleton /// membrane /// integral to membrane	---
207390_s_at	-5.184	1.924E-04	SMTN	smoothelin	smooth muscle contraction /// muscle development	actin binding /// structural constituent of muscle /// structural molecule activity	actin cytoskeleton /// cytoskeleton	---
203370_s_at	-6.02	5.612E-05	PDLIM7	PDZ and LIM domain 7 (enigma)	ossification /// cell differentiation /// development	protein binding /// zinc ion binding /// metal ion binding /// protein binding	cytoskeleton	---

204400_at	-7.093	1.412E-05	EFS	embryonal Fyn-associated substrate	cell adhesion /// intracellular signaling cascade	protein binding	cytoplasm	---
204083_s_at	-6.067	5.213E-05	TPM2	tropomyosin 2 (beta)	---	actin binding /// structural constituent of muscle /// structural constituent of cytoskeleton	cytoskeleton /// muscle thin filament tropomyosin	Striated_muscle_contraction
200878_at	-5.269	1.681E-04	EPAS1	endothelial PAS domain protein 1	angiogenesis /// response to hypoxia /// regulation of transcription, DNA-dependent /// transcription from RNA polymerase II promoter /// signal transduction /// cell differentiation /// transcription /// signal transduction /// development /// regulation of transcription	RNA polymerase II transcription factor activity, enhancer binding /// transcription coactivator activity /// signal transducer activity /// protein binding /// histone acetyltransferase binding /// DNA binding /// transcription factor activity /// transcription regulator activity	nucleus	---
218831_s_at	-5.198	1.884E-04	FCGRT	Fc fragment of IgG, receptor, transporter, alpha	pregnancy /// antigen presentation /// immune response /// antigen presentation, endogenous antigen /// antigen processing, endogenous antigen via MHC class I /// immune response	receptor activity /// IgG binding /// MHC class I receptor activity /// receptor activity	membrane /// integral to membrane /// MHC class I protein complex	---
200911_s_at	-6.232	4.242E-05	TACC1	transforming, acidic coiled-coil containing protein 1	cell cycle /// cell division	protein binding	nucleus	---

201425_at	-6.214	4.242E-05	ALDH2	aldehyde dehydrogenase 2 family (mitochondrial)	carbohydrate metabolism /// alcohol metabolism /// metabolism	aldehyde dehydrogenase (NAD) activity /// aldehyde dehydrogenase [NAD(P)+] activity /// electron carrier activity /// aldehyde dehydrogenase (NAD) activity /// oxidoreductase activity	mitochondrion	Glycolysis / Gluconeogenesis /// Ascorbate and aldarate metabolism /// Fatty acid metabolism /// Bile acid biosynthesis /// Valine, leucine and isoleucine degradation /// Lysine degradation /// Arginine and proline metabolism /// Histidine metabolism /// Tryptophan metabolism /// beta-Alanine metabolism /// Glycerolipid metabolism /// Pyruvate metabolism /// 1,2-Dichloroethane degradation /// Propanoate metabolism /// Butanoate metabolism /// Limonene and pinene degradation
213698_at	-5.51	1.187E-04	ZMYM6	zinc finger, MYM-type 6	development	DNA binding /// zinc ion binding /// metal ion binding	nucleus	---
205026_at	-5.131	2.121E-04	STAT5B	signal transducer and activator of transcription 5B	transcription /// regulation of transcription from RNA polymerase II promoter /// intracellular signaling cascade /// JAK-STAT cascade /// regulation of transcription, DNA-dependent /// signal transduction	transcription factor activity /// signal transducer activity /// calcium ion binding /// DNA binding /// transcription factor activity	nucleus	---

823_at	-7.509	8.324E-06	CX3CL1	chemokine (C-X3-C motif) ligand 1	immune response /// cell adhesion /// cytokine and chemokine mediated signaling pathway /// immune cell chemotaxis /// positive regulation of inflammatory response /// leukocyte adhesive activation /// positive regulation of calcium-independent cell-cell adhesion /// immune response /// defense response	chemokine activity /// protein binding /// cytokine activity /// protein binding /// chemokine activity /// receptor binding	extracellular region /// extracellular space /// cell surface /// membrane /// integral to membrane /// extracellular region /// integral to membrane	---
203892_at	-6.393	3.469E-05	WFDC2	WAP four-disulfide core domain 2	proteolysis /// spermatogenesis	serine-type endopeptidase inhibitor activity /// endopeptidase inhibitor activity /// endopeptidase inhibitor activity	extracellular space	---
218574_s_at	-5.961	5.829E-05	LMCD1	LIM and cysteine-rich domains 1	---	zinc ion binding /// metal ion binding	---	---
201667_at	-5.173	1.969E-04	GJA1	gap junction protein, alpha 1, 43kDa (connexin 43)	transport /// muscle contraction /// cell-cell signaling /// heart development /// sensory perception of sound /// gap junction assembly /// positive regulation of I-kappaB kinase/NF-kappaB cascade /// cell communication	signal transducer activity /// protein binding /// ion transporter activity /// connexon channel activity /// connexon channel activity	integral to plasma membrane /// connexon complex /// membrane /// gap junction /// connexon complex /// integral to membrane	Calcium_regulation_in_cardiac_cells /// Smooth_muscle_contraction
201389_at	-5.098	2.256E-04	ITGA5	integrin, alpha 5 (fibronectin receptor, alpha polypeptide)	cell adhesion /// integrin-mediated signaling pathway /// cell adhesion	receptor activity /// calcium ion binding /// protein binding	ruffle /// integrin complex /// membrane /// integral to membrane /// integrin complex	Integrin-mediated_cell_adhesion_KEGG
205251_at	-5.487	1.220E-04	PER2	period homolog 2 (Drosophila)	transcription /// regulation of transcription, DNA-dependent /// signal transduction /// circadian rhythm /// rhythmic process	signal transducer activity	nucleus	Circadian_Exercise /// Circadian_Exercise

203979_at	-6.546	2.777E-05	CYP27A1	cytochrome P450, family 27, subfamily A, polypeptide 1	electron transport	iron ion binding /// steroid hydroxylase activity /// heme binding /// metal ion binding /// cholestanetriol 26-monooxygenase activity /// monooxygenase activity /// oxidoreductase activity	mitochondrion /// membrane /// mitochondrion	Bile acid biosynthesis /// PPAR signaling pathway
203810_at	-5.554	1.108E-04	DNAJB4	DnaJ (Hsp40) homolog, subfamily B, member 4	protein folding /// response to unfolded protein /// response to heat /// response to unfolded protein	heat shock protein binding /// unfolded protein binding /// unfolded protein binding /// protein binding	---	---
202762_at	-5.083	2.308E-04	ROCK2	Rho-associated, coiled-coil containing protein kinase 2	cytokinesis /// protein amino acid phosphorylation /// muscle contraction /// intracellular signaling cascade /// protein amino acid phosphorylation /// signal transduction	nucleotide binding /// protein serine/threonine kinase activity /// structural molecule activity /// ATP binding /// zinc ion binding /// transferase activity /// diacylglycerol binding /// metal ion binding /// protein kinase activity /// protein serine/threonine kinase activity /// kinase activity	intracellular /// actin cytoskeleton	G13_Signaling_Pathway /// Integrin-mediated_cell_adhesion _KEGG
218683_at	-5.9	6.275E-05	PTBP2	polypyrimidine tract binding protein 2	nuclear mRNA splicing, via spliceosome /// mRNA processing	nucleotide binding /// RNA binding /// nucleic acid binding	nucleus	mRNA_processing_Rea ctome
209337_at	-5.176	1.960E-04	PSIP1	PC4 and SFRS1 interacting protein 1	transcription /// regulation of transcription, DNA-dependent	DNA binding	nucleus	---
204422_s_at	-6.546	2.777E-05	FGF2	fibroblast growth factor 2 (basic)	regulation of progression through cell cycle /// activation of MAPK activity /// angiogenesis /// chemotaxis /// signal transduction /// Ras protein signal transduction /// cell-cell signaling /// nervous system development /// muscle development /// cell proliferation /// positive regulation of cell proliferation /// organ morphogenesis /// cell differentiation /// development	protein binding /// growth factor activity /// heparin binding	extracellular region /// extracellular space	---

200713_s_at	-6.106	4.934E-05	MAPRE1	microtubule-associated protein, RP/EB family, member 1	regulation of progression through cell cycle /// cell cycle /// mitosis /// cell proliferation /// negative regulation of microtubule polymerization /// cell division	protein C-terminus binding /// microtubule plus-end binding /// protein binding /// microtubule binding	spindle /// microtubule /// cortical microtubule cytoskeleton	---
204134_at	-5.5	1.204E-04	PDE2A	phosphodiesterase 2A, cGMP-stimulated	signal transduction	catalytic activity /// 3',5'-cyclic-nucleotide phosphodiesterase activity /// cGMP-stimulated cyclic-nucleotide phosphodiesterase activity /// hydrolase activity	membrane	---
201092_at	-5.639	9.755E-05	RBBP7	retinoblastoma binding protein 7	DNA replication /// transcription /// regulation of transcription, DNA-dependent /// development /// cell proliferation /// chromatin modification	protein binding	nucleus	---
210202_s_at	-5.104	2.240E-04	BIN1	bridging integrator 1	cell cycle /// cell proliferation /// regulation of endocytosis /// cell differentiation /// negative regulation of progression through cell cycle /// endocytosis /// development /// ATP biosynthesis /// transport /// ion transport /// ATP synthesis coupled proton transport /// proton transport /// regulation of progression through cell cycle /// generation of precursor metabolites and energy	protein binding /// hydrogen ion transporter activity /// hydrolase activity /// metal ion binding /// hydrogen-transporting ATP synthase activity, rotational mechanism /// hydrogen-transporting ATPase activity, rotational mechanism /// transporter activity	nucleus /// cytoplasm /// actin cytoskeleton /// mitochondrion /// membrane /// proton-transporting two-sector ATPase complex /// proton-transporting ATP synthase complex, catalytic core F(1) /// proton-transporting ATP synthase complex (sensu Eukaryota) /// membrane fraction	Electron_Transport_Chain /// Electron_Transport_Chain
38157_at	5.348	1.505E-04	DOM3Z	dom-3 homolog Z (C. elegans)	---	protein binding /// identical protein binding	---	---

205412_at	5.849	6.877E-05	ACAT1	acetyl-Coenzyme A acetyltransferase 1 (acetoacetyl Coenzyme A thiolase)	---	acetyl-CoA C-acetyltransferase activity /// acyltransferase activity /// transferase activity /// acetyl-CoA C-acetyltransferase activity	mitochondrion /// mitochondrion	Synthesis_and_Degradation_of_Keton_Bodies_KEGG /// Synthesis_and_Degradation_of_Keton_Bodies_KEGG /// Fatty acid metabolism /// Synthesis and degradation of ketone bodies /// Valine, leucine and isoleucine degradation /// Lysine degradation /// Tryptophan metabolism /// Pyruvate metabolism /// Benzoate degradation via CoA ligation /// Propanoate metabolism /// Butanoate metabolism /// Two-component system - General
219725_at	5.028	2.467E-04	TREM2	triggering receptor expressed on myeloid cells 2	humoral immune response	receptor activity /// receptor activity	membrane /// integral to membrane	---
213553_x_at	5.208	1.850E-04	APOC1	apolipoprotein C-I	lipid metabolism /// lipid transport /// lipoprotein metabolism /// transport	lipid transporter activity	extracellular region	Statin_Pathway_PharmGKB
213892_s_at	5.235	1.772E-04	APRT	adenine phosphoribosyltransferase	purine ribonucleoside salvage /// adenine salvage /// nucleoside metabolism	adenine phosphoribosyltransferase activity /// AMP binding /// transferase activity, transferring glycosyl groups /// adenine phosphoribosyltransferase activity /// transferase activity	cytoplasm	Purine metabolism

205937_at	5.146	2.063E-04	CGREF1	cell growth regulator with EF-hand domain 1	response to stress /// cell cycle /// cell cycle arrest /// negative regulation of cell proliferation	calcium ion binding /// kinase activity	---	---
211576_s_at	7.458	8.362E-06	SLC19A1	solute carrier family 19 (folate transporter), member 1	transport /// folic acid transport	folic acid binding /// folic acid transporter activity /// reduced folate carrier activity /// methotrexate transporter activity /// nutrient reservoir activity	membrane fraction /// integral to plasma membrane /// membrane /// integral to membrane	---
202148_s_at	8.161	4.812E-06	PYCR1	pyrroline-5-carboxylate reductase 1	electron transport /// proline biosynthesis /// proline biosynthesis /// amino acid biosynthesis	pyrroline-5-carboxylate reductase activity /// pyrroline-5-carboxylate reductase activity /// oxidoreductase activity	---	Urea cycle and metabolism of amino groups /// Arginine and proline metabolism
211558_s_at	6.036	5.529E-05	DHPS	deoxyhypusine synthase	protein biosynthesis /// positive regulation of cell proliferation /// hypusine biosynthesis from peptidyl-lysine /// spermidine catabolism to deoxyhypusine, using deoxyhypusine synthase /// spermidine catabolism to deoxyhypusine, using deoxyhypusine synthase	transferase activity	---	---
217736_s_at	6.922	1.797E-05	EIF2AK1	eukaryotic translation initiation factor 2-alpha kinase 1	response to stress /// response to external stimulus /// negative regulation of translational initiation by iron /// protein amino acid autophosphorylation /// negative regulation of hemoglobin biosynthesis /// protein biosynthesis /// protein amino acid phosphorylation /// negative regulation of protein biosynthesis	nucleotide binding /// eukaryotic translation initiation factor 2alpha kinase activity /// ATP binding /// transferase activity /// heme binding /// protein homodimerization activity /// protein kinase activity /// protein serine/threonine kinase activity /// kinase activity /// damaged DNA binding	---	Translation_Factors

201923_at	7.957	5.782E-06	PRDX4	peroxiredoxin 4	I-kappaB phosphorylation	thioredoxin peroxidase activity /// oxidoreductase activity /// peroxiredoxin activity /// peroxidase activity /// antioxidant activity	---	---
65517_at	6.457	3.219E-05	AP1M2	adaptor-related protein complex 1, mu 2 subunit	protein targeting /// vesicle targeting /// transport /// intracellular protein transport /// protein transport	---	coated pit /// clathrin vesicle coat	---
202740_at	5.371	1.471E-04	ACY1	aminoacylase 1	proteolysis /// amino acid metabolism /// amino acid metabolism	aminoacylase activity /// metallopeptidase activity /// zinc ion binding /// hydrolase activity /// protein dimerization activity /// aminoacylase activity	cytoplasm /// cytosol	Urea cycle and metabolism of amino groups
201622_at	5.505	1.202E-04	SND1	staphylococcal nuclease domain containing 1	transcription /// regulation of transcription, DNA-dependent	nucleic acid binding /// transcription cofactor activity /// nuclease activity	nucleus /// nucleus	---
218387_s_at	5.95	5.829E-05	PGLS	6-phosphogluconolactonase	carbohydrate metabolism /// pentose-phosphate shunt /// pentose-phosphate shunt	hydrolase activity /// 6-phosphogluconolactonase activity	---	Pentose_Phosphate_Pathway /// Pentose phosphate pathway
220587_s_at	5.515	1.181E-04	GBL	G protein beta subunit-like	---	---	---	---
218275_at	6.713	2.237E-05	SLC25A10	solute carrier family 25 (mitochondrial carrier; dicarboxylate transporter), member 10	gluconeogenesis /// transport /// dicarboxylic acid transport /// mitochondrial transport	dicarboxylic acid transporter activity /// binding	mitochondrion /// membrane /// integral to membrane	---
212429_s_at	5.751	8.288E-05	GTF3C2	general transcription factor IIIC, polypeptide 2, beta 110kDa	transcription /// transcription from RNA polymerase III promoter	RNA polymerase III transcription factor activity /// protein binding	transcription factor TFIIIC complex /// nucleus	---
200807_s_at	5.924	6.104E-05	HSPD1	heat shock 60kDa protein 1 (chaperonin)	protein folding /// response to unfolded protein /// protein import into mitochondrial matrix /// regulation of apoptosis /// cellular protein metabolism /// protein folding	nucleotide binding /// protein binding /// ATP binding /// ATP binding /// unfolded protein binding /// chaperone binding /// unfolded protein binding	cytoplasm /// mitochondrion /// mitochondrion	---
218680_x_at	5.624	9.940E-05	HYPK	Huntingtin interacting protein K	---	protein binding	---	---

209036_s_at	5.054	2.372E-04	MDH2	malate dehydrogenase 2, NAD (mitochondrial)	glycolysis /// tricarboxylic acid cycle /// malate metabolism /// tricarboxylic acid cycle intermediate metabolism	L-lactate dehydrogenase activity /// oxidoreductase activity /// L-malate dehydrogenase activity /// malate dehydrogenase activity /// L-malate dehydrogenase activity	mitochondrion /// mitochondrial matrix	Glycolysis_and_Gluconeogenesis /// Krebs-TCA_Cycle /// Citrate cycle (TCA cycle) /// Pyruvate metabolism /// Glyoxylate and dicarboxylate metabolism /// Carbon fixation /// Reductive carboxylate cycle (CO2 fixation)
203663_s_at	6.01	5.652E-05	COX5A	cytochrome c oxidase subunit Va	electron transport	cytochrome-c oxidase activity /// iron ion binding /// electron carrier activity /// metal ion binding /// cytochrome-c oxidase activity /// oxidoreductase activity	mitochondrion /// membrane	Electron_Transport_Chain /// Electron_Transport_Chain /// Oxidative phosphorylation /// VEGF signaling pathway
208756_at	6.947	1.797E-05	EIF3S2	eukaryotic translation initiation factor 3, subunit 2 beta, 36kDa	protein biosynthesis /// regulation of translational initiation	translation initiation factor activity /// protein binding /// translation factor activity, nucleic acid binding	eukaryotic translation initiation factor 3 complex	Translation_Factors /// Translation_Factors
220741_s_at	5.032	2.458E-04	PPA2 /// RNF36	pyrophosphatase (inorganic) 2 /// ring finger protein 36	phosphate metabolism	magnesium ion binding /// inorganic diphosphatase activity /// hydrolase activity /// protein binding /// zinc ion binding /// metal ion binding	cytoplasm /// mitochondrion	Oxidative phosphorylation
202785_at	5.389	1.436E-04	NDUFA7	NADH dehydrogenase (ubiquinone) 1 alpha subcomplex, 7, 14.5kDa	ATP synthesis coupled electron transport	NADH dehydrogenase activity /// NADH dehydrogenase (ubiquinone) activity /// oxidoreductase activity /// NADH dehydrogenase (ubiquinone) activity	membrane fraction /// mitochondrion /// mitochondrial inner membrane	Electron_Transport_Chain
201698_s_at	5.229	1.779E-04	SFRS9	splicing factor, arginine/serine-rich 9	nuclear mRNA splicing, via spliceosome /// mRNA splice site selection /// mRNA processing /// mRNA processing	nucleotide binding /// RNA binding /// nucleic acid binding	nucleus /// nucleus	mRNA_processing_Reactome

200044_at	5.097	2.256E-04	SFRS9	splicing factor, arginine/serine-rich 9 /// splicing factor, arginine/serine-rich 9	nuclear mRNA splicing, via spliceosome /// mRNA splice site selection /// mRNA processing /// mRNA processing	nucleotide binding /// RNA binding /// nucleic acid binding	nucleus /// nucleus	mRNA_processing_Reactome
217740_x_at	6.202	4.269E-05	RPL7A	ribosomal protein L7a	protein biosynthesis /// ribosome biogenesis and assembly	structural constituent of ribosome	intracellular /// ribosome /// ribonucleoprotein complex	Ribosomal_Proteins /// Ribosomal_Proteins /// Ribosomal_Proteins
200809_x_at	5.446	1.311E-04	RPL12	ribosomal protein L12	protein biosynthesis /// phosphate transport	RNA binding /// structural constituent of ribosome /// RNA binding /// structural constituent of ribosome	intracellular /// ribosome /// cytosolic large ribosomal subunit (sensu Eukaryota) /// ribonucleoprotein complex /// cytoplasm /// extracellular matrix (sensu Metazoa)	Ribosomal_Proteins
208764_s_at	5.098	2.256E-04	ATP5G2	ATP synthase, H+ transporting, mitochondrial F0 complex, subunit C2 (subunit 9)	ion transport /// ATP synthesis coupled proton transport /// proton transport /// transport	transporter activity /// lipid binding /// hydrogen-transporting ATP synthase activity, rotational mechanism /// hydrogen-transporting ATPase activity, rotational mechanism /// hydrogen ion transporter activity	membrane fraction /// mitochondrion /// proton-transporting ATP synthase complex (sensu Eukaryota) /// membrane /// proton-transporting two-sector ATPase complex /// proton-transporting ATP synthase complex, coupling factor F(o) /// integral to membrane	Electron_Transport_Chain /// Electron_Transport_Chain /// Oxidative phosphorylation /// ATP synthesis /// Photosynthesis /// Flagellar assembly /// Type III secretion system /// Epithelial cell signaling in Helicobacter pylori /// Purine metabolism
202857_at	5.952	5.829E-05	TMEM4	transmembrane protein 4	---	protein binding	integral to plasma membrane /// endoplasmic reticulum	---
201256_at	5.36	1.492E-04	COX7A2L	cytochrome c oxidase subunit VIIa polypeptide 2 like	electron transport	cytochrome-c oxidase activity /// electron carrier activity /// cytochrome-c oxidase activity	mitochondrion /// mitochondrial electron transport chain	Electron_Transport_Chain
200819_s_at	5.158	2.021E-04	RPS15	ribosomal protein S15	protein biosynthesis /// protein biosynthesis /// metal ion transport	structural constituent of ribosome /// protein binding /// structural constituent of ribosome /// metal ion transporter activity	intracellular /// cytosolic small ribosomal subunit (sensu Eukaryota) /// ribosome /// small ribosomal subunit /// ribonucleoprotein complex /// membrane	Ribosomal_Proteins /// Ribosomal_Proteins /// Ribosomal_Proteins /// Ribosomal_Proteins

218226_s_at	5.272	1.679E-04	NDUFB4 /// LOC653432	NADH dehydrogenase (ubiquinone) 1 beta subcomplex, 4, 15kDa /// similar to NADH dehydrogenase (ubiquinone) 1 beta subcomplex, 4, 15kDa	electron transport /// mitochondrial electron transport, NADH to ubiquinone	NADH dehydrogenase activity /// NADH dehydrogenase (ubiquinone) activity /// NADH dehydrogenase (ubiquinone) activity /// oxidoreductase activity	membrane fraction /// mitochondrion	Electron_Transport_Chain /// Electron_Transport_Chain /// Oxidative phosphorylation /// Ubiquinone biosynthesis /// Oxidative phosphorylation
218025_s_at	5.051	2.374E-04	PECI	peroxisomal D3,D2-enoyl-CoA isomerase	fatty acid metabolism /// metabolism	acyl-CoA binding /// dodecenoyl-CoA delta-isomerase activity /// isomerase activity /// catalytic activity	peroxisome /// peroxisome	---
212694_s_at	6.72	2.237E-05	PCCB	propionyl Coenzyme A carboxylase, beta polypeptide	fatty acid catabolism	propionyl-CoA carboxylase activity /// ligase activity /// propionyl-CoA carboxylase activity	mitochondrion /// mitochondrion	Valine, leucine and isoleucine degradation /// Propanoate metabolism
209426_s_at	8.374	3.758E-06	AMACR	alpha-methylacyl-CoA racemase	metabolism /// phosphate transport	alpha-methylacyl-CoA racemase activity /// lyase activity /// isomerase activity /// catalytic activity	mitochondrion /// peroxisome /// cytoplasm /// mitochondrion /// peroxisome	---
206858_s_at	7.226	1.180E-05	HOXC6	homeobox C6	regulation of transcription, DNA-dependent /// regulation of transcription from RNA polymerase II promoter /// development /// transcription /// development /// regulation of transcription /// regulation of transcription, DNA-dependent /// development	transcription factor activity /// transcription corepressor activity /// sequence-specific DNA binding /// DNA binding /// transcriptional activator activity /// transcription factor activity	nucleus /// nucleus	---
202941_at	5.571	1.088E-04	NDUFV2	NADH dehydrogenase (ubiquinone) flavoprotein 2, 24kDa	mitochondrial electron transport, NADH to ubiquinone /// nervous system development /// mitochondrial electron transport, NADH to ubiquinone	NADH dehydrogenase activity /// iron ion binding /// NADH dehydrogenase (ubiquinone) activity /// metal ion binding /// 2 iron, 2 sulfur cluster binding /// NADH dehydrogenase (ubiquinone) activity /// oxidoreductase activity /// iron-sulfur cluster binding /// electron carrier activity	membrane fraction /// mitochondrion	Electron_Transport_Chain /// Oxidative phosphorylation /// Ubiquinone biosynthesis /// Oxidative phosphorylation

211474_s_at	5.443	1.315E-04	SERPINB6	serpin peptidase inhibitor, clade B (ovalbumin), member 6	---	serine-type endopeptidase inhibitor activity /// serine-type endopeptidase inhibitor activity /// protein binding /// endopeptidase inhibitor activity	cytosol	---
201135_at	5.264	1.692E-04	ECHS1	enoyl Coenzyme A hydratase, short chain, 1, mitochondrial	generation of precursor metabolites and energy /// lipid metabolism /// fatty acid metabolism /// fatty acid beta-oxidation /// metabolism	enoyl-CoA hydratase activity /// lyase activity /// catalytic activity /// enoyl-CoA hydratase activity	mitochondrion /// mitochondrion	Fatty acid elongation in mitochondria /// Fatty acid metabolism /// Valine, leucine and isoleucine degradation /// Lysine degradation /// Tryptophan metabolism /// beta-Alanine metabolism /// Benzoate degradation via CoA ligation /// Propanoate metabolism /// Butanoate metabolism /// Limonene and pinene degradation /// Caprolactam degradation /// PPAR signaling pathway
219041_s_at	5.289	1.636E-04	REPIN1	replication initiator 1	DNA replication	DNA binding /// zinc ion binding /// metal ion binding /// nucleic acid binding	intracellular /// nucleus /// nuclear origin of replication recognition complex	---
201527_at	5.021	2.485E-04	ATP6V1F	ATPase, H+ transporting, lysosomal 14kDa, V1 subunit F	ion transport /// ATP synthesis coupled proton transport /// proton transport /// ATP biosynthesis /// transport /// proton transport	hydrolase activity /// metal ion binding /// hydrogen-transporting ATP synthase activity, rotational mechanism /// hydrogen-transporting ATPase activity, rotational mechanism /// hydrogen ion transporter activity /// hydrogen ion transporter activity	membrane fraction /// proton-transporting two-sector ATPase complex /// proton-transporting two-sector ATPase complex	Oxidative phosphorylation /// ATP synthesis /// Photosynthesis /// Flagellar assembly /// Type III secretion system /// Epithelial cell signaling in Helicobacter pylori

204478_s_at	5.526	1.160E-04	RABIF	RAB interacting factor	membrane fusion /// small GTPase mediated signal transduction /// protein transport /// transport	guanyl-nucleotide exchange factor activity /// zinc ion binding /// metal ion binding /// guanyl-nucleotide exchange factor activity /// zinc ion binding /// guanyl-nucleotide exchange factor activity	---	---
213812_s_at	5.523	1.164E-04	CAMKK2	calcium/calmodulin-dependent protein kinase kinase 2, beta	MAPKKK cascade /// calcium-mediated signaling /// regulation of protein kinase activity /// positive regulation of transcription /// protein amino acid autophosphorylation /// protein amino acid phosphorylation /// protein amino acid phosphorylation /// protein amino acid phosphorylation	nucleotide binding /// protein serine/threonine kinase activity /// calcium- and calmodulin-dependent protein kinase activity /// protein-tyrosine kinase activity /// calcium ion binding /// calmodulin binding /// ATP binding /// transferase activity /// protein kinase activity /// protein serine/threonine kinase activity /// calmodulin binding /// ATP binding /// kinase activity	intracellular	---
205645_at	5.671	9.484E-05	REPS2	RALBP1 associated Eps domain containing 2	protein complex assembly /// epidermal growth factor receptor signaling pathway	calcium ion binding /// calcium ion binding /// protein binding	---	---
200895_s_at	5.402	1.397E-04	FKBP4	FK506 binding protein 4, 59kDa	protein folding /// protein folding	peptidyl-prolyl cis-trans isomerase activity /// FK506 binding /// isomerase activity /// protein binding, bridging /// protein binding /// binding	intracellular /// nucleus /// cytoplasm	Calcium signaling pathway
209265_s_at	5.686	9.278E-05	METTL3	methyltransferase like 3	RNA methylation /// nucleobase, nucleoside, nucleotide and nucleic acid metabolism	RNA binding /// methyltransferase activity /// mRNA (2'-O-methyladenosine-N6-)-methyltransferase activity /// transferase activity /// mRNA (2'-O-methyladenosine-N6-)-methyltransferase activity	nucleus /// nucleus	mRNA_processing_Reactome

206558_at	6.795	2.065E-05	SIM2	single-minded homolog 2 (Drosophila)	regulation of transcription, DNA-dependent /// signal transduction /// nervous system development /// cell differentiation /// transcription /// development /// nervous system development /// regulation of transcription	transcription factor activity /// signal transducer activity /// DNA binding /// transcription factor activity /// transcription regulator activity /// DNA binding	nucleus	---
207949_s_at	5.292	1.636E-04	ICA1	islet cell autoantigen 1, 69kDa	neurotransmitter transport /// regulation of neurotransmitter secretion /// transport	---	Golgi membrane /// cytoplasm /// cytosol /// membrane /// secretory granule membrane /// synaptic vesicle membrane	---
204319_s_at	5.914	6.118E-05	RGS10	regulator of G-protein signalling 10	negative regulation of signal transduction	signal transducer activity	---	Calcium_regulation_in_cardiac_cells /// Calcium_regulation_in_cardiac_cells /// Smooth_muscle_contraction /// Smooth_muscle_contraction
204973_at	5.495	1.209E-04	GJB1	gap junction protein, beta 1, 32kDa (connexin 32, Charcot-Marie-Tooth neuropathy, X-linked)	transport /// cell-cell signaling /// nervous system development /// cell communication	connexon channel activity /// connexon channel activity	connexon complex /// integral to membrane /// gap junction /// connexon complex	Calcium_regulation_in_cardiac_cells
200644_at	7.782	6.745E-06	MARCKSL1	MARCKS-like 1	---	calmodulin binding	---	---
203243_s_at	5.371	1.471E-04	PDLIM5	PDZ and LIM domain 5	heart development	actin binding /// protein kinase C binding /// protein binding /// zinc ion binding /// receptor signaling complex scaffold activity /// actinin binding /// metal ion binding	membrane fraction /// cytosol	---
201078_at	5.413	1.377E-04	TM9SF2	transmembrane 9 superfamily member 2	transport /// transport	transporter activity	endosome /// integral to plasma membrane /// membrane /// integral to membrane	---
205780_at	5.024	2.484E-04	BIK	BCL2-interacting killer (apoptosis-inducing)	induction of apoptosis /// apoptotic program /// regulation of apoptosis /// apoptosis	protein binding	membrane /// integral to membrane	---
204667_at	5.092	2.271E-04	FOXA1	forkhead box A1	transcription /// regulation of transcription, DNA-dependent	transcription factor activity /// sequence-specific DNA binding /// DNA binding /// transcription factor activity	nucleus	---

209696_at	7.487	8.324E-06	FBP1	fructose-1,6-bisphosphatase 1	carbohydrate metabolism /// fructose metabolism /// gluconeogenesis	fructose-2,6-bisphosphate 2-phosphatase activity /// zinc ion binding /// hydrolase activity /// fructose-bisphosphatase activity /// phosphoric ester hydrolase activity /// identical protein binding /// catalytic activity	---	Glycolysis_and_Gluconeogenesis /// Glycolysis / Gluconeogenesis /// Pentose phosphate pathway /// Fructose and mannose metabolism /// Carbon fixation /// Insulin signaling pathway
203196_at	7.148	1.310E-05	ABCC4	ATP-binding cassette, sub-family C (CFTR/MRP), member 4	ion transport /// transport	nucleotide binding /// chloride channel activity /// ATP binding /// 15-hydroxyprostaglandin dehydrogenase (NAD+) activity /// ATPase activity /// ATPase activity, coupled to transmembrane movement of substances /// nucleoside-triphosphatase activity	membrane fraction /// plasma membrane /// integral to membrane /// platelet dense granule membrane /// membrane	---
201839_s_at	8.533	3.175E-06	TACSTD1	tumor-associated calcium signal transducer 1	---	---	plasma membrane /// integral to membrane /// membrane	---
200971_s_at	6.255	4.215E-05	SERP1	stress-associated endoplasmic reticulum protein 1	protein modification /// protein amino acid glycosylation /// response to stress /// plasma membrane organization and biogenesis	---	endoplasmic reticulum /// ribosome	---
208737_at	5.422	1.358E-04	ATP6V1G1	ATPase, H+ transporting, lysosomal 13kDa, V1 subunit G1	ATP biosynthesis /// ion transport /// proton transport /// transport	hydrogen ion transporter activity /// hydrolase activity /// metal ion binding	---	Oxidative phosphorylation /// ATP synthesis /// Photosynthesis /// Flagellar assembly /// Type III secretion system /// Epithelial cell signaling in Helicobacter pylori
203857_s_at	5.489	1.220E-04	PDIA5	protein disulfide isomerase family A, member 5	electron transport /// protein folding /// response to stress	protein disulfide isomerase activity /// electron carrier activity /// protein disulfide oxidoreductase activity /// isomerase activity /// protein disulfide isomerase activity /// oxidoreductase activity	endoplasmic reticulum /// endoplasmic reticulum lumen	Antigen processing and presentation

218313_s_at	5.031	2.458E-04	GALNT7	UDP-N-acetyl-alpha-D-galactosamine:polypeptide N-acetylgalactosaminyltransferase 7 (GalNAc-T7)	carbohydrate metabolism /// protein amino acid O-linked glycosylation	polypeptide N-acetylgalactosaminyltransferase activity /// calcium ion binding /// sugar binding /// transferase activity, transferring glycosyl groups /// manganese ion binding /// transferase activity	membrane /// integral to membrane	O-Glycan biosynthesis /// Glycan structures - biosynthesis 1
200656_s_at	6.279	4.069E-05	P4HB	procollagen-proline, 2-oxoglutarate 4-dioxygenase (proline 4-hydroxylase), beta polypeptide	protein folding /// peptidyl-proline hydroxylation to 4-hydroxy-L-proline	protein disulfide isomerase activity /// procollagen-proline 4-dioxygenase activity /// electron carrier activity /// protein disulfide oxidoreductase activity /// isomerase activity /// unfolded protein binding /// protein disulfide isomerase activity /// procollagen-proline 4-dioxygenase activity	extracellular region /// endoplasmic reticulum /// ER-Golgi intermediate compartment /// cell surface /// membrane /// endoplasmic reticulum	Arginine and proline metabolism /// Antigen processing and presentation
217771_at	5.488	1.220E-04	GOLPH2	golgi phosphoprotein 2	---	---	Golgi apparatus /// integral to plasma membrane /// membrane /// integral to membrane	---
201900_s_at	6.004	5.675E-05	AKR1A1	aldo-keto reductase family 1, member A1 (aldehyde reductase)	glucose metabolism /// aldehyde metabolism	aldehyde reductase activity /// protein binding /// alcohol dehydrogenase (NADP+) activity /// electron carrier activity /// oxidoreductase activity	---	Glycolysis / Gluconeogenesis /// Glycerolipid metabolism /// Caprolactam degradation
219736_at	5.163	2.003E-04	TRIM36	tripartite motif-containing 36	chitin metabolism	protein binding /// zinc ion binding /// metal ion binding /// chitin binding	intracellular /// extracellular region	---
205757_at	5.344	1.515E-04	ENTPD5	ectonucleoside triphosphate diphosphohydrolase 5	---	magnesium ion binding /// calcium ion binding /// hydrolase activity /// nucleoside-diphosphatase activity	endoplasmic reticulum /// membrane /// integral to membrane	---
203911_at	6.309	3.885E-05	RAP1GAP	RAP1 GTPase activating protein	signal transduction /// signal transduction /// signal transduction	GTPase activity /// GTPase activator activity /// GTPase activator activity	membrane fraction /// cytosol /// membrane	---
201886_at	5.471	1.252E-04	WDR23	WD repeat domain 23	---	---	---	---

217979_at	5.249	1.730E-04	TSPAN13	Tetraspanin 13	---	---	membrane fraction /// integral to plasma membrane /// membrane /// integral to membrane	---
201079_at	6.173	4.441E-05	SYNGR2	synaptogyrin 2	---	---	integral to plasma membrane /// membrane /// integral to membrane	---
208837_at	5.647	9.755E-05	TMED3	transmembrane emp24 protein transport domain containing 3	protein transport /// transport	---	membrane /// integral to membrane	---
204934_s_at	9.121	1.317E-06	HPN	hepsin (transmembrane protease, serine 1)	proteolysis	serine-type endopeptidase activity /// scavenger receptor activity /// peptidase activity /// hydrolase activity /// serine-type peptidase activity	integral to plasma membrane /// membrane /// integral to membrane	---
210243_s_at	5.567	1.089E-04	B4GALT3	UDP-Gal:betaGlcNAc beta 1,4-galactosyltransferase, polypeptide 3	carbohydrate metabolism	beta-N-acetylglucosaminylglycopeptide beta-1,4-galactosyltransferase activity /// N-acetyllactosamine synthase activity /// galactosyltransferase activity /// transferase activity, transferring glycosyl groups /// manganese ion binding /// metal ion binding /// transferase activity	membrane /// integral to membrane	N-Glycan biosynthesis /// Keratan sulfate biosynthesis /// Glycan structures - biosynthesis 1
209665_at	6.114	4.871E-05	CYB561D2	cytochrome b-561 domain containing 2	electron transport /// transport	iron ion binding /// metal ion binding	membrane /// integral to membrane	---
220161_s_at	5.797	7.525E-05	EPB41L4B	erythrocyte membrane protein band 4.1 like 4B	---	structural constituent of cytoskeleton /// binding /// cytoskeletal protein binding	cytoplasm /// cytoskeleton /// cytoskeleton /// membrane	---
209238_at	5.641	9.755E-05	STX3	syntaxin 3	neurotransmitter transport /// intracellular protein transport /// transport	protein transporter activity	membrane /// integral to membrane	---

201413_at	5.051	2.374E-04	HSD17B4	hydroxysteroid (17-beta) dehydrogenase 4	lipid metabolism /// fatty acid metabolism /// metabolism	3-hydroxyacyl-CoA dehydrogenase activity /// estradiol 17-beta-dehydrogenase activity /// sterol carrier activity /// sterol transporter activity /// oxidoreductase activity /// lyase activity /// isomerase activity /// catalytic activity /// sterol carrier activity	peroxisome /// peroxisome	Steroid_Biosynthesis
222212_s_at	6.013	5.652E-05	LASS2	LAG1 longevity assurance homolog 2 (S. cerevisiae)	regulation of transcription, DNA-dependent /// lipid biosynthesis /// regulation of transcription	transcription factor activity /// sequence-specific DNA binding /// DNA binding	nucleus /// endoplasmic reticulum /// membrane /// integral to membrane	---
204088_at	5.408	1.389E-04	P2RX4	purinergic receptor P2X, ligand-gated ion channel, 4	ion transport /// signal transduction /// transport /// transport	receptor activity /// ATP-gated cation channel activity /// ion channel activity /// ATP binding	integral to plasma membrane /// membrane /// integral to membrane	---
209504_s_at	5.063	2.351E-04	PLEKHB1	pleckstrin homology domain containing, family B (evectins) member 1	phototransduction	signal transducer activity	membrane /// integral to membrane	---
202605_at	5.14	2.072E-04	GUSB	glucuronidase, beta	carbohydrate metabolism /// glycosaminoglycan catabolism /// carbohydrate metabolism /// glycosaminoglycan metabolism	beta-glucuronidase activity /// cation binding /// catalytic activity /// hydrolase activity, hydrolyzing O-glycosyl compounds /// beta-glucuronidase activity /// hydrolase activity /// hydrolase activity, acting on glycosyl bonds	lysosome /// lysosome	Pentose and glucuronate interconversions /// Starch and sucrose metabolism /// Glycosaminoglycan degradation /// Porphyrin and chlorophyll metabolism /// Glycan structures - degradation
218931_at	5.742	8.387E-05	RAB17	RAB17, member RAS oncogene family	small GTPase mediated signal transduction /// protein transport /// transport	nucleotide binding /// GTP binding	membrane	---

200652_at	5.063	2.351E-04	SSR2	signal sequence receptor, beta (translocon-associated protein beta)	cotranslational protein targeting to membrane /// protein biosynthesis	signal sequence binding /// protein binding /// structural constituent of ribosome /// structural constituent of ribosome /// receptor activity	endoplasmic reticulum /// endoplasmic reticulum /// membrane /// integral to membrane /// integral to membrane /// cytosolic small ribosomal subunit (sensu Eukaryota) /// intracellular /// ribosome /// ribonucleoprotein complex	Ribosomal_Proteins /// Ribosomal_Proteins
207414_s_at	5.213	1.839E-04	PCSK6	proprotein convertase subtilisin/kexin type 6	proteolysis /// proteolysis /// cell-cell signaling	subtilase activity /// subtilase activity /// calcium ion binding /// peptidase activity /// serine-type endopeptidase activity /// hydrolase activity	endoplasmic reticulum	---
217912_at	5.406	1.393E-04	DUS1L	dihydrouridine synthase 1-like (S. cerevisiae)	tRNA processing	oxidoreductase activity /// FAD binding	---	---
208658_at	5.259	1.712E-04	PDIA4	protein disulfide isomerase family A, member 4	protein secretion	protein disulfide isomerase activity /// calcium ion binding /// electron carrier activity /// protein disulfide oxidoreductase activity /// isomerase activity	endoplasmic reticulum /// endoplasmic reticulum lumen	---
203430_at	5.894	6.328E-05	HEBP2	heme binding protein 2	---	---	---	---
203216_s_at	6.241	4.242E-05	MYO6	myosin VI	striated muscle contraction /// sensory perception of sound /// actin filament-based movement /// sensory perception of sound	microfilament motor activity /// nucleotide binding /// motor activity /// actin binding /// calmodulin binding /// ATP binding /// ATP binding /// structural constituent of muscle /// ATPase activity, coupled /// actin binding /// structural molecule activity /// motor activity	unconventional myosin /// unconventional myosin /// myosin	---
202939_at	5.964	5.829E-05	ZMPSTE24	zinc metallopeptidase (STE24 homolog, yeast)	proteolysis /// proteolysis	metalloendopeptidase activity /// metalloexopeptidase activity /// zinc ion binding /// metal ion binding /// peptidase activity /// metallopeptidase activity /// hydrolase activity	endoplasmic reticulum /// membrane /// integral to membrane	---

200863_s_at	7.612	7.551E-06	RAB11A	RAB11A, member RAS oncogene family	regulation of transcription, DNA-dependent /// small GTPase mediated signal transduction /// protein transport /// plasma membrane to endosome transport /// transport	nucleotide binding /// GTPase activity /// transporter activity /// ATP binding /// GTP binding /// transcription factor binding /// syntaxin binding /// protein binding	intracellular /// Golgi trans face	---
209494_s_at	5.964	5.829E-05	ZNF278	zinc finger protein 278	transcription /// regulation of transcription, DNA-dependent /// regulation of transcription, DNA-dependent	DNA binding /// protein binding /// zinc ion binding /// transcriptional repressor activity /// metal ion binding /// nucleic acid binding /// DNA binding	intracellular /// nucleus /// nucleus	---
200057_s_at	5.387	1.440E-04	NONO	non-POU domain containing, octamer-binding /// non-POU domain containing, octamer-binding	nuclear mRNA splicing, via spliceosome /// DNA repair /// DNA recombination /// transcription /// regulation of transcription, DNA-dependent /// RNA splicing /// mRNA processing /// response to DNA damage stimulus /// mRNA processing	nucleotide binding /// DNA binding /// RNA binding /// protein binding /// nucleic acid binding	nucleus /// nucleus	mRNA_processing_Reactome /// mRNA_processing_Reactome
208695_s_at	5.328	1.546E-04	RPL39	ribosomal protein L39	protein biosynthesis	structural constituent of ribosome	intracellular /// ribosome /// ribonucleoprotein complex	Ribosomal_Proteins /// Ribosomal_Proteins /// mRNA_processing_binding_Reactome /// Ribosomal_Proteins
208591_s_at	5.355	1.492E-04	PDE3B	phosphodiesterase 3B, cGMP-inhibited	signal transduction	catalytic activity /// 3',5'-cyclic-nucleotide phosphodiesterase activity /// cGMP-inhibited cyclic-nucleotide phosphodiesterase activity /// hydrolase activity	membrane	---
206949_s_at	5.687	9.278E-05	RUSC1	RUN and SH3 domain containing 1	---	SH3/SH2 adaptor activity	nucleus	---
206572_x_at	7.223	1.180E-05	ZNF85	zinc finger protein 85	transcription /// regulation of transcription, DNA-dependent	nucleic acid binding /// transcription factor activity /// transcription corepressor activity /// zinc ion binding /// metal ion binding /// DNA binding /// DNA binding	intracellular /// nucleus /// nucleus	---

219664_s_at	5.314	1.597E-04	DECR2	2,4-dienoyl CoA reductase 2, peroxisomal	metabolism /// GTP biosynthesis /// UTP biosynthesis /// CTP biosynthesis /// nucleotide metabolism /// nucleoside metabolism	2,4-dienoyl-CoA reductase (NADPH) activity /// oxidoreductase activity /// nucleotide binding /// magnesium ion binding /// nucleoside diphosphate kinase activity /// ATP binding /// kinase activity /// transferase activity /// metal ion binding /// nucleoside diphosphate kinase activity	peroxisome /// mitochondrion /// mitochondrion	---
219061_s_at	5.114	2.193E-04	LAGE3	L antigen family, member 3	---	---	---	---
220160_s_at	5.416	1.377E-04	KPTN	kaptin (actin binding protein)	cell motility /// actin filament organization /// blood coagulation /// sensory perception of sound	actin binding /// actin binding	nucleus /// microtubule organizing center /// actin cytoskeleton	---
219708_at	5.057	2.372E-04	NT5M	5',3'-nucleotidase, mitochondrial	DNA replication /// nucleotide metabolism /// pyrimidine deoxyribonucleotide catabolism	nucleotide binding /// magnesium ion binding /// nucleotidase activity /// hydrolase activity /// metal ion binding	mitochondrion /// mitochondrion	Purine metabolism /// Pyrimidine metabolism /// Nicotinate and nicotinamide metabolism
208457_at	5.332	1.540E-04	GABRD	gamma-aminobutyric acid (GABA) A receptor, delta	ion transport /// signal transduction /// transport /// transport	GABA-A receptor activity /// ion channel activity /// extracellular ligand-gated ion channel activity /// chloride channel activity /// neurotransmitter receptor activity /// chloride ion binding /// GABA-A receptor activity	integral to plasma membrane /// postsynaptic membrane /// membrane /// integral to membrane	---
210746_s_at	5.096	2.256E-04	EPB42	erythrocyte membrane protein band 4.2 /// erythrocyte membrane protein band 4.2	regulation of cell shape /// peptide cross-linking /// erythrocyte maturation	structural constituent of cytoskeleton /// ATP binding /// structural molecule activity	cytoskeleton /// plasma membrane /// cytoskeleton	---
221300_at	6.308	3.885E-05	C15orf2	chromosome 15 open reading frame 2	spermatogenesis /// cell differentiation	---	---	---
209622_at	5.741	8.387E-05	STK16	serine/threonine kinase 16	protein complex assembly /// protein amino acid phosphorylation /// protein amino acid phosphorylation	nucleotide binding /// protein serine/threonine kinase activity /// ATP binding /// transferase activity /// protein kinase activity /// protein serine/threonine kinase activity /// kinase activity	membrane	---

204228_at	5.278	1.665E-04	PPIH	peptidylprolyl isomerase H (cyclophilin H)	nuclear mRNA splicing, via spliceosome /// protein folding /// protein complex assembly /// snRNP protein import into nucleus /// mRNA processing /// protein folding	peptidyl-prolyl cis-trans isomerase activity /// cyclosporin A binding /// isomerase activity /// unfolded protein binding	nucleus /// spliceosome complex /// spliceosome complex	---
218145_at	5.857	6.877E-05	TRIB3	tribbles homolog 3 (Drosophila)	transcription /// regulation of transcription, DNA-dependent /// protein amino acid phosphorylation /// negative regulation of protein kinase activity /// apoptosis /// regulation of MAPK activity /// apoptosis	transcription corepressor activity /// protein kinase activity /// protein kinase inhibitor activity /// protein binding /// protein binding /// ATP binding /// protein kinase binding /// protein kinase binding /// kinase activity	nucleus /// nucleus	---
209635_at	5.331	1.543E-04	AP1S1	adaptor-related protein complex 1, sigma 1 subunit	intracellular protein transport /// receptor mediated endocytosis /// vesicle-mediated transport /// transport /// intracellular protein transport /// endocytosis /// protein transport	protein binding /// protein transporter activity	Golgi trans face /// coated pit /// AP-1 adaptor complex /// AP-1 adaptor complex /// coated vesicle membrane /// clathrin coat of trans-Golgi network vesicle	---
209825_s_at	5.158	2.021E-04	UCK2	uridine-cytidine kinase 2	biosynthesis	nucleotide binding /// uridine kinase activity /// ATP binding /// kinase activity /// transferase activity	---	Pyrimidine metabolism

201268_at	5.635	9.791E-05	NME2 /// NME1-NME2	non-metastatic cells 2, protein (NM23B) expressed in /// NM23-LV	GTP biosynthesis /// UTP biosynthesis /// CTP biosynthesis /// transcription /// regulation of transcription, DNA-dependent /// cell cycle /// cell adhesion /// negative regulation of cell proliferation /// nucleotide metabolism /// nucleoside triphosphate biosynthesis /// negative regulation of progression through cell cycle /// regulation of transcription, DNA-dependent /// pyrimidine ribonucleoside triphosphate biosynthesis	nucleotide binding /// magnesium ion binding /// transcription factor activity /// nucleoside diphosphate kinase activity /// protein binding /// ATP binding /// kinase activity /// transferase activity /// DNA binding /// nucleoside diphosphate kinase activity /// ATP binding /// metal ion binding	ruffle /// nucleus /// lamellipodium /// nucleus	Nucleotide_Metabolism /// Purine metabolism /// Pyrimidine metabolism
203228_at	5.61	1.019E-04	PAFAH1B3	platelet-activating factor acetylhydrolase, isoform Ib, gamma subunit 29kDa	nervous system development /// lipid catabolism /// lipid metabolism /// lipid metabolism /// central nervous system development	1-alkyl-2-acetylglycerophosphocholine esterase activity /// protein binding /// hydrolase activity, acting on ester bonds /// hydrolase activity /// growth factor activity /// growth factor activity	soluble fraction /// cytoplasm /// membrane /// extracellular region	---
202159_at	5.065	2.351E-04	FARSLA	phenylalanine-tRNA synthetase-like, alpha subunit	protein biosynthesis /// phenylalanyl-tRNA aminoacylation /// tRNA aminoacylation for protein translation /// protein biosynthesis /// phenylalanyl-tRNA aminoacylation	nucleotide binding /// phenylalanine-tRNA ligase activity /// ATP binding /// ligase activity /// aminoacyl-tRNA ligase activity /// phenylalanine-tRNA ligase activity	soluble fraction /// cytoplasm /// cytoplasm	---

201577_at	5.292	1.636E-04	NME1	non-metastatic cells 1, protein (NM23A) expressed in	GTP biosynthesis /// UTP biosynthesis /// CTP biosynthesis /// cell cycle /// negative regulation of cell proliferation /// nucleotide metabolism /// nucleoside triphosphate biosynthesis /// regulation of apoptosis /// negative regulation of progression through cell cycle /// pyrimidine ribonucleoside triphosphate biosynthesis /// transcription /// regulation of transcription, DNA-dependent /// regulation of transcription, DNA-dependent /// cell adhesion	nucleotide binding /// magnesium ion binding /// magnesium ion binding /// DNA binding /// deoxyribonuclease activity /// nucleoside diphosphate kinase activity /// nucleoside diphosphate kinase activity /// ATP binding /// ATP binding /// kinase activity /// transferase activity /// protein binding /// metal ion binding /// DNA binding /// transcription factor activity /// nucleoside diphosphate kinase activity	nucleus /// nucleus /// cytoplasm /// ruffle /// lamellipodium /// nucleus	Nucleotide_Metabolism /// Purine metabolism /// Pyrimidine metabolism
201479_at	5.256	1.717E-04	DKC1	dyskeratosis congenita 1, dyskerin	regulation of progression through cell cycle /// rRNA processing /// telomere maintenance via telomerase /// cell proliferation /// rRNA processing /// RNA processing /// ribosome biogenesis /// RNA processing	telomerase activity /// RNA binding /// pseudouridylate synthase activity /// isomerase activity /// RNA binding	nucleus /// nucleoplasm /// telomerase holoenzyme complex /// nucleolus /// ribonucleoprotein complex	---
222216_s_at	5.023	2.484E-04	MRPL17	mitochondrial ribosomal protein L17	protein biosynthesis	structural constituent of ribosome	intracellular /// mitochondrion /// ribosome /// ribonucleoprotein complex	---
218188_s_at	5.553	1.108E-04	TIMM13	translocase of inner mitochondrial membrane 13 homolog (yeast)	protein folding /// sensory perception of sound /// protein transport /// protein import into mitochondrial inner membrane /// protein amino acid ADP-ribosylation /// chromatin silencing /// regulation of transcription, DNA-dependent /// protein targeting /// protein targeting to mitochondrion /// transport /// protein targeting to mitochondrion	zinc ion binding /// metal ion binding /// unfolded protein binding /// NAD(P)+-protein-arginine ADP-ribosyltransferase activity /// zinc ion binding /// DNA binding /// NAD(P)+-protein-arginine ADP-ribosyltransferase activity /// zinc ion binding /// transferase activity /// transferase activity, transferring glycosyl groups	mitochondrion /// mitochondrial inner membrane presequence translocase complex /// membrane /// mitochondrial intermembrane space protein transporter complex /// nucleus /// nucleus /// chromatin silencing complex	---

202144_s_at	6.624	2.517E-05	ADSL	adenylosuccinate lyase	purine ribonucleotide biosynthesis /// purine nucleotide biosynthesis	adenylosuccinate lyase activity /// lyase activity /// catalytic activity /// adenylosuccinate lyase activity	---	Nucleotide_Metabolism /// Purine metabolism /// Alanine and aspartate metabolism
201013_s_at	7.005	1.637E-05	PAICS	phosphoribosylaminoimidazole carboxylase, phosphoribosylaminoimidazole succinocarboxamide synthetase	purine nucleotide biosynthesis /// 'de novo' IMP biosynthesis /// purine base biosynthesis	phosphoribosylaminoimidazole carboxylase activity /// phosphoribosylaminoimidazolesuccinocarboxamide synthase activity /// ATP binding /// lyase activity /// ligase activity /// identical protein binding /// catalytic activity /// phosphoribosylaminoimidazolesuccinocarboxamide synthase activity /// carboxy-lyase activity	phosphoribosylaminoimidazole carboxylase complex	Purine metabolism /// Purine metabolism
201391_at	5.83	7.131E-05	TRAP1	TNF receptor-associated protein 1	protein folding /// response to unfolded protein	nucleotide binding /// tumor necrosis factor receptor binding /// ATP binding /// unfolded protein binding /// receptor activity	mitochondrion	---
201115_at	5.055	2.372E-04	POLD2	polymerase (DNA directed), delta 2, regulatory subunit 50kDa	DNA replication /// DNA replication	DNA binding /// DNA-directed DNA polymerase activity /// protein binding /// transferase activity /// nucleotidyltransferase activity	nucleus /// nucleus	DNA_replication_Reactome /// Purine metabolism /// Pyrimidine metabolism /// DNA polymerase
208972_s_at	5.802	7.484E-05	ATP5G1	ATP synthase, H+ transporting, mitochondrial F0 complex, subunit C1 (subunit 9)	ion transport /// ATP synthesis coupled proton transport /// proton transport /// transport	transporter activity /// lipid binding /// hydrogen-transporting ATP synthase activity, rotational mechanism /// hydrogen-transporting ATPase activity, rotational mechanism /// hydrogen ion transporter activity	membrane fraction /// mitochondrion /// proton-transporting ATP synthase complex (sensu Eukaryota) /// membrane /// proton-transporting two-sector ATPase complex /// proton-transporting ATP synthase complex, coupling factor F(o) /// integral to membrane /// mitochondrial inner membrane	Electron_Transport_Chain /// Oxidative phosphorylation /// ATP synthesis /// Photosynthesis /// Flagellar assembly /// Type III secretion system /// Epithelial cell signaling in Helicobacter pylori /// Purine metabolism

205512_s_at	5.664	9.537E-05	PDCD8	programmed cell death 8 (apoptosis-inducing factor)	electron transport /// DNA fragmentation during apoptosis /// apoptosis /// DNA damage response, signal transduction resulting in induction of apoptosis	DNA binding /// protein binding /// electron carrier activity /// disulfide oxidoreductase activity /// oxidoreductase activity /// FAD binding	nucleus /// mitochondrion /// nucleus /// mitochondrion	---
203867_s_at	5.123	2.153E-04	NLE1	notchless homolog 1 (Drosophila)	---	---	nucleus	---
204247_s_at	5.964	5.829E-05	CDK5	cyclin-dependent kinase 5	protein amino acid phosphorylation /// cell cycle /// striated muscle development /// cell proliferation /// embryonic development /// neuron differentiation /// neurite development /// positive regulation of neuron apoptosis /// axon extension /// cell division	nucleotide binding /// cyclin-dependent protein kinase activity /// ErbB-2 class receptor binding /// protein binding /// protein binding /// ATP binding /// transferase activity /// acetylcholine receptor activator activity /// ErbB-3 class receptor binding /// tau-protein kinase activity /// protein serine/threonine kinase activity /// kinase activity /// protein kinase activity /// protein serine/threonine kinase activity /// kinase activity /// protein kinase activity /// protein serine/threonine kinase activity	nucleus /// cytoplasm /// membrane /// axon /// dendrite /// growth cone /// neuromuscular junction /// cell soma	---
200826_at	5.067	2.351E-04	SNRPD2	small nuclear ribonucleoprotein D2 polypeptide 16.5kDa	nuclear mRNA splicing, via spliceosome /// mRNA processing /// mRNA metabolism	protein binding	nucleus /// ribonucleoprotein complex	mRNA_processing_Reactome /// mRNA_processing_binding_Reactome
201931_at	5.081	2.308E-04	ETFA	electron-transfer-flavoprotein, alpha polypeptide (glutaric aciduria II)	electron transport /// transport	electron carrier activity /// FAD binding /// electron carrier activity	mitochondrion /// mitochondrial matrix /// mitochondrion	---
218455_at	5.215	1.838E-04	NFS1	NFS1 nitrogen fixation 1 (S. cerevisiae)	sulfur amino acid metabolism /// protein complex assembly /// metabolism	transaminase activity /// transferase activity /// cysteine desulfurase activity	nucleus /// mitochondrion /// cytosol /// mitochondrion	---
218557_at	5.67	9.484E-05	NIT2	nitrilase family, member 2	nitrogen compound metabolism	hydrolase activity, acting on carbon-nitrogen (but not peptide) bonds	---	---

208864_s_at	5.547	1.117E-04	TXN	thioredoxin	electron transport /// cell motility /// signal transduction /// cell-cell signaling /// cell proliferation /// transport	protein binding /// electron carrier activity /// protein disulfide oxidoreductase activity /// thiol-disulfide exchange intermediate activity	---	---
204649_at	5.577	1.083E-04	TROAP	trophinin associated protein (tastin)	cell adhesion /// cell adhesion	protein binding	cytoplasm	---
204033_at	6.494	3.019E-05	TRIP13	thyroid hormone receptor interactor 13	transcription from RNA polymerase II promoter	nucleotide binding /// transcription cofactor activity /// ATP binding /// nucleoside-triphosphatase activity	nucleus	---
203947_at	5.644	9.755E-05	CSTF3	cleavage stimulation factor, 3' pre-RNA, subunit 3, 77kDa	mRNA polyadenylylation /// mRNA cleavage /// RNA processing /// mRNA processing	RNA binding /// binding	intracellular /// nucleus /// nucleus	mRNA_processing_Reactome /// mRNA_processing_Reactome
