# Supplementary material for: Co-regulation analysis of closely linked genes identifies a highly recurrent gain on chromosome 17q25.3 in prostate cancer
Source: BMC Cancer. 2008 Oct 30;8:315. doi: 10.1186/1471-2407-8-315 (PMC2585097; doi:10.1186/1471-2407-8-315)
Supplement: Additional file 2 — Graphical representation of the genes selected by FADA. Unsupervised hierarchical clusters built with the expression data from the 318 genes selected by FADA as the most discriminant genes between tumoral and normal samples. [file 1471-2407-8-315-S2.pdf]

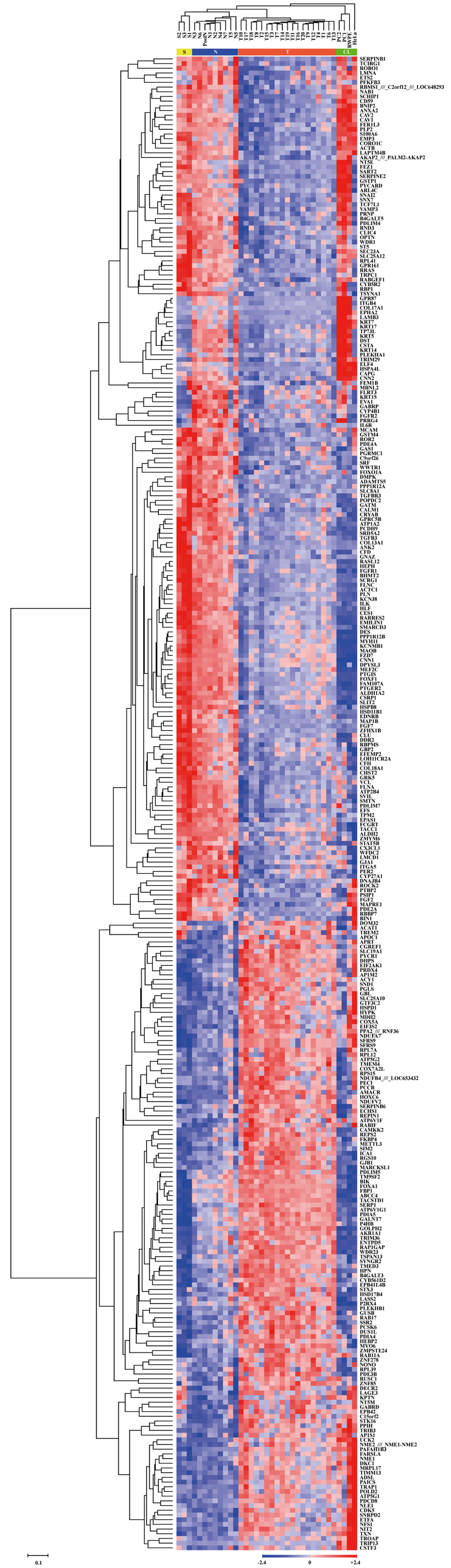

S2
S3
S1
N3
N6
PoolN
N1
N2
N4
N7
T5
N5
T10
T17
T18
T8
T2
T15
T3
T7
T14
T19
T11
T16
T20
T9
T12
T4
T1
T6
T13
PC3
PC1
RWPE1
HeLa
S
N
T
CL
SERPINB1
TCIRG1
ROBO1
LMNA
ETS2
PFKFB3
RBMS1_///_C2orf12_///_LOC648293
NAB1
SCHIP1
CD59
BNIP2
ANXA2
CAV2
CAV1
FER1L3
PLP2
S100A6
EMP3
CORO1C
ACTB
LAPTM4B
AKAP2_///_PALM2-AKAP2
NT5E
FEZ1
SART2
SERPINE2
GSTP1
PYCARD
ARL4C
SNAI2
SNX7
TCF7L1
VAMP3
PRNP
B4GALT5
PDLIM4
RND3
CLIC4
OPTN
WDR1
ST5
SEC23A
SLC25A12
RPL41
GPR161
RRAS
TRPC1
RABGEF1
CYB5R2
RBP1
TSYNA1
GPR87
ITGB4
COL17A1
EPHA2
LAMB3
KRT7
KRT17
TP73L
KRT5
DST
CSTA
KRT14
PLEKHA1
TRIM29
ELF4
HSPA4L
CAPG
CNN2
FEM1B
MBNL2
FLRT3
KRT15
EVA1
GABRP
CYP4B1
FGFR2
PRRG4
IL6R
MCAM
GSTM4
ROR2
PDE4A
GAS1
PGRMC1
C9orf26
SRF
WWTR1
FOXO1A
DMPK
ADAMTS5
PPP1R12A
SLC8A1
TGFBR3
POPDC2
GATM
CALM1
CRYAB
GPRC5B
ATP1A2
PCDH9
SRD5A2
TGFB3
COL13A1
ANK2
CFD
GNAZ
RASL12
HEPH
FGFR1
BHMT2
SCRG1
FLNC
ACTC1
PLN
KCNJ8
ILK
HLF
CES1
RARRES2
EMILIN1
SMARCD3
DES
PPP1R12B
MYH11
KCNMB1
MAOB
FZD7
CNN1
DPYSL3
MEF2C
PTGIS
FOXF1
FAM107A
PTGER2
ALDH1A2
CSRP1
SLIT2
HSPB8
HSD11B1
EDNRB
MAP1B
FGF7
ZFHX1B
CLU
DDR2
RBPMS
GBP2
EFEMP2
LOH11CR2A
CFH
COL18A1
CHST2
GRK5
VCL
FLNA
ATP2B4
SVIL
SMTN
PDLIM7
EFS
TPM2
EPAS1
FCGRT
TACC1
ALDH2
ZMYM6
STAT5B
CX3CL1
WFDC2
LMCD1
GJA1
ITGA5
PER2
CYP27A1
DNAJB4
ROCK2
PTBP2
PSIP1
FGF2
MAPRE1
PDE2A
RBBP7
BIN1
DOM32
ACAT1
TREM2
APOC1
APRT
CGREF1
SLC19A1
PYCR1
DHPS
EIF2AK1
PRDX4
AP1M2
ACY1
SND1
PGLS
GBL
SLC25A10
GTF3C2
HSPD1
HYPK
MDH2
COX5A
EIF3S2
PPA2_///_RNF36
NDUFA7
SFRS9
SFRS9
RPL7A
RPL12
ATP5G2
TMEM4
COX7A2L
RPS15
NDUFB4_///_LOC653432
PEC1
PCCB
AMACR
HOXC6
NDUFV2
SERPINB6
ECHS1
REPIN1
ATP6V1F
RAB1F
CAMKK2
REPS2
FKBP4
METTL3
SIM2
ICA1
RGS10
GJB1
MARCKSL1
PDLIM5
TM9SF2
BIK
FOXA1
FBP1
ABCC4
TACSTD1
SERP1
ATP6V1G1
PDIA5
GALNT7
P4HB
GOLPH2
AKR1A1
TRIM36
ENTPD5
RAP1GAP
WDR23
TSPAN13
SYNGR2
TMED3
HPN
B4GALT3
CYB561D2
EPB41L4B
STX3
HSD17B4
LASS2
P2RX4
PLEKHB1
GUSB
RAB17
SSR2
PCSK6
DUS1L
PDIA4
HEBP2
MYO6
ZMPSTE24
RAB11A
ZNF278
NONO
RPL39
PDE3B
RUSC1
ZNF85
DECR2
LAGE3
KPTN
NT5M
GABRD
EPB42
C15orf2
STK16
PPIH
TRIB3
AP1S1
UCK2
NME2_///_NME1-NME2
PAFAH1B3
FARSLA
NME1
DKC1
MRPL17
TIMM13
ADSL
PAICS
TRAP1
POLD2
ATP5G1
PDCD8
NLE1
CDK5
SNRPD2
ETFA
NFS1
NIT2
TXN
TROAP
TRIP13
CSTF3
0.1
-2.4
0
+2.4
